# LIFU/MMP-2 dual-responsive release of repurposed drug disulfiram from nanodroplets for inhibiting vasculogenic mimicry and lung metastasis in triple-negative breast cancer

**DOI:** 10.1186/s12951-024-02492-7

**Published:** 2024-04-25

**Authors:** Ying Liu, Rui Tang, Yuting Cao, Nianhong Wu, Qiaoxi Qin, Yuanyuan Chen, Xi Wei, Jianli Ren, Yang Sun, Hong Zhou, Yang Zhou, Pan Li

**Affiliations:** 1 Department of Ultrasound, The Second Affiliated Hospital of Chongqing Medical University, Institute of Ultrasound Imaging of Chongqing Medical University, Chongqing, China; 2 Department of Ultrasound, The Second Affiliated Hospital of Chongqing Medical University, State Key Laboratory of Ultrasound in Medicine and Engineering of Chongqing Medical University, No.76 Linjiang Road, Yuzhong District, Chongqing, 400010 China; 3 Department of Ultrasound, The Third People’s Hospital of Chengdu City, The Affiliated Hospital of Southwest Jiaotong University, No. 82 Qinglong Street, Chengdu, 610031 Sichuan China; 4 Department of Pathology, The Third People’s Hospital of Chengdu City, The Affiliated Hospital of Southwest Jiaotong University, Chengdu, China; 5 Department of Diagnostic and Therapeutic Ultrasonography, Tianjin Medical University Cancer Institute and Hospital, National Clinical Research Center for Cancer, Key Laboratory of Cancer Prevention and Therapy, Tianjin’s Clinical Research Center for Cancer, Tianjin, China

**Keywords:** Vasculogenic mimicry, Matrix metalloproteinase-2, Low-intensity focused ultrasound, Disulfiram, Drug penetration

## Abstract

**Background:**

Vasculogenic mimicry (VM), when microvascular channels are formed by cancer cells independent of endothelial cells, often occurs in deep hypoxic areas of tumors and contributes to the aggressiveness and metastasis of triple-negative breast cancer (TNBC) cells. However, well-developed VM inhibitors exhibit inadequate efficacy due to their low drug utilization rate and limited deep penetration. Thus, a cost-effective VM inhibition strategy needs to be designed for TNBC treatment.

**Results:**

Herein, we designed a low-intensity focused ultrasound (LIFU) and matrix metalloproteinase-2 (MMP-2) dual-responsive nanoplatform termed PFP@PDM-PEG for the cost-effective and efficient utilization of the drug disulfiram (DSF) as a VM inhibitor. The PFP@PDM-PEG nanodroplets effectively penetrated tumors and exhibited substantial accumulation facilitated by PEG deshielding in a LIFU-mediated and MMP-2-sensitive manner. Furthermore, upon exposure to LIFU irradiation, DSF was released controllably under ultrasound imaging guidance. This secure and controllable dual-response DSF delivery platform reduced VM formation by inhibiting COL1/pro-MMP-2 activity, thereby significantly inhibiting tumor progression and metastasis.

**Conclusions:**

Considering the safety of the raw materials, controlled treatment process, and reliable repurposing of DSF, this dual-responsive nanoplatform represents a novel and effective VM-based therapeutic strategy for TNBC in clinical settings.

**Graphical Abstract:**

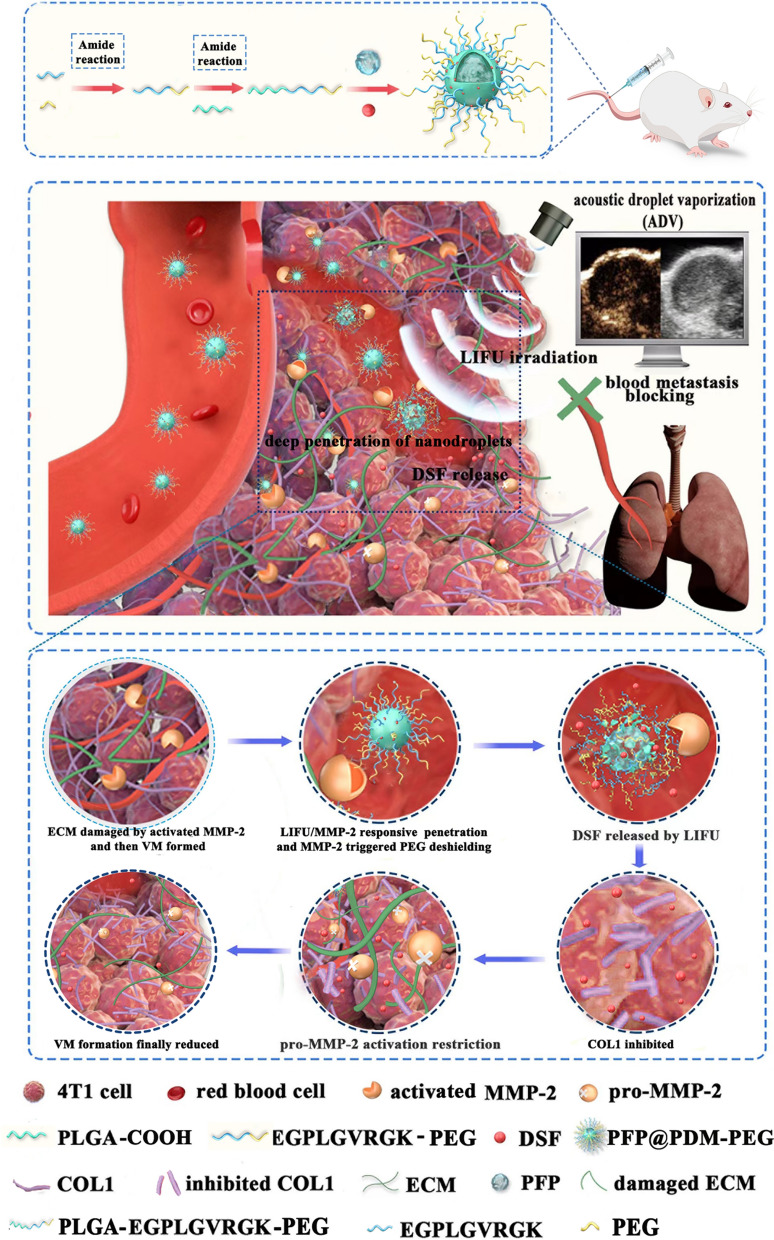

**Supplementary Information:**

The online version contains supplementary material available at 10.1186/s12951-024-02492-7.

## Introduction

Vasculogenic mimicry (VM), when tube-like structures are formed by cancer cells independent of endothelial cells, is a distinct pattern of tumor angiogenesis for tumor growth, especially in the hypoxic central region [1–4]. These capillary-like structures are composed of endothelial tumor cells and are supported by periodic acid–Schiff (PAS)-positive cells and a rich extracellular matrix (ECM), which delivers nutrients and oxygen and accelerates tumor growth [5, 6]. VM is associated with high tumor grade, accelerated proliferation, and poor prognosis in patients with different malignant tumors, including triple-negative breast cancer (TNBC) [7, 8]. The absence of an endothelial lining in VM also provides tumor cells with greater access to circulation, thereby accelerating tumor metastasis [9, 10]. Moreover, VM can lead to the failure of multiple antitumor therapies, including antiangiogenic agents, chemotherapies, and immunotherapies [3, 11]. Therefore, developing a safe and efficient treatment strategy by inhibiting VM will greatly improve the clinical outcome of cancer patients.

A series of developed inhibitors have been applied for antitumor therapy based on multiple mechanisms of VM formation, including VE-Cad, Ras, HER2, VEGF, COX-2, and the MMP-2 pathway [12, 13]. Extensive research and development costs have been dedicated to such inhibition. Despite the impressive results from in vitro experiments and animal models, the effectiveness of these inhibitors for broad clinical translation remains unsatisfactory [14–16]. The increased areas of VM tend to concentrate in the central regions of the tumor where severe hypoxia is prevalent, which results in extremely low utilization of these carefully developed inhibitors [4, 17]. In addition, the dense extracellular matrix (ECM) hinders the penetration and uniform distribution of inhibitors. Finally, balancing toxicity/side effects with the efficacy of systematic administration are also major problems that should be considered in the development of VM inhibitors. Therefore, the development of cost-effective VM inhibitors to safely and efficiently inhibit tumor recurrence and metastasis is urgently needed.

Repurposing of existing drugs is a safe, reliable, and cost-effective approach for developing VM inhibitors. For instance, disulfiram (DSF), which was originally used for chronic alcoholism, was recently found to regulate the differentiation of tumor stem cells and promote apoptosis by reshaping energy metabolism through acetaldehyde dehydrogenase (ALDH), an NAD^+^-dependent protein [18–21]. More importantly, DSF can reduce the expression of type I collagen (COL1, the main component of the ECM) and affect precursor matrix metalloproteinase-2 (pro-MMP-2) activation [21, 22]. The decrease in COL1 expression could reduce the MT1-MMP-mediated activation of MMP-2 by increasing MT1-MMP mRNA and protein levels [23–27]. Inhibition of activated MMP-2 could also reduce the degradation of the basement membrane within the ECM, thereby inhibiting VM and preventing distant metastasis [23, 28]. This study provides a valuable opportunity for the cost-effective development and efficient utilization of VM inhibitors in clinical settings. However, DSF is unstable in acidic environments and rapidly degrades in the bloodstream, resulting in relatively low bioavailability [29, 30]. Thus, a well-designed drug delivery platform needs to be further developed to deliver DSF to TNBC, especially in deep areas where VM is prone to occur.

Due to the rapid development of nanotechnology, low-intensity focused ultrasound (LIFU)/MMP-2 dual-responsive nanoplatforms are expected to promote the deep penetration and uniform dispersion of bioavailable DSF in TNBC. Matrix metalloproteinases (MMPs) are significantly overexpressed in deep hypoxic areas hypoxia, where VM is prone to occur, compared to their lower levels in normal tissues [31, 32]. Therefore, MMP-2-responsive dePEGylation was utilized to effectively inhibit VM formation in deep areas. Given the specifically high level of MMP-2 where VM is prone to occur, the PEG shed in response to MMP-2 provides a great impetus for DSF uptake by specific cancer cells [33–36]. Moreover, LIFU-responsive phase change nanodroplets, mainly including lipids and polymers, could serve as noninvasive and precise tools for ultrasound (US) imaging-guided DSF delivery. More importantly, the liquid‒gas phase change of LIFU-responsive nanodroplets can disrupt the dense ECM, improve deep tumor penetration, and distribute DSF uniformly through the cavitation effect and enhance penetration [37–39].

**Scheme 1:**
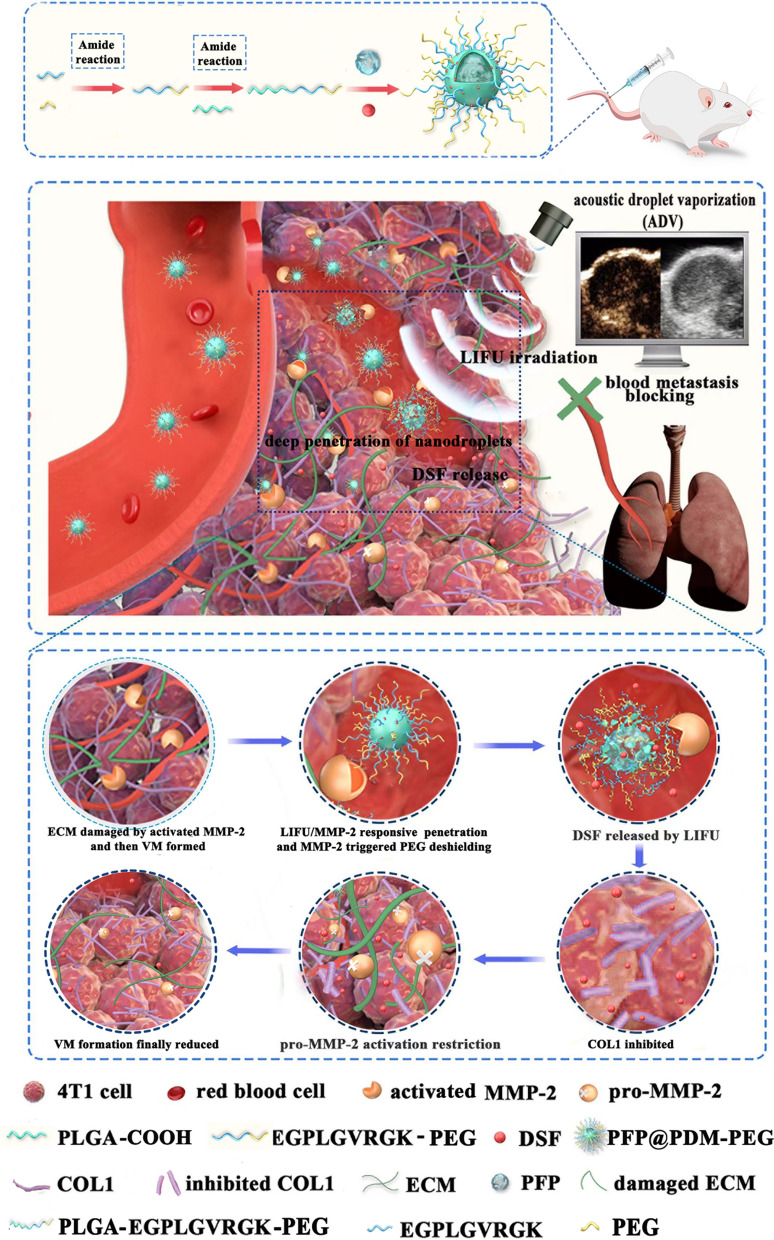
Schematic of the phase transition of PFP@PDM-PEG for efficient targeted inhibition of VM and pulmonary metastasis in triple-negative breast cancer

As illustrated in Scheme 1, we designed and assembled a LIFU/MMP-2 dual-responsive and phase-changeable nanoplatform loaded with DSF for VM-based cancer therapy. PLGA-MMP-2-PEG was first synthesized with an MMP-2 substrate peptide and then used to encapsulate perfluoropentane (PFP) and load DSF to generate a nanodroplet (designated PFP@PDM-PEG) as an ultrasound molecular probe for drug delivery visualization. PFP@PDM-PEG efficiently accumulated in tumor tissues where VM is prone to occur and then the PEG group was shed in response to hydrolysis by MMP-2 present in high levels in the tumor ECM, leading to enhanced tumor cell uptake. When subjected to LIFU irradiation, the nanodroplets underwent a conversion into microbubbles. This conversion resulted in enhanced ultrasound imaging performance, facilitated on-demand DSF release, and improved penetration and distribution of DSF. The DSF released from PFP@PDM-PEG reduced VM formation by inhibiting COL1 expression, thereby affecting pro-MMP-2 activation, which further inhibited tumor growth and distant metastasis. The design strategy of this LIFU/MMP-2 dual-responsive nanoplatform offers a promising approach for VM-based TNBC treatment by repurposing DSF, potentially leading to cost savings and expediting the clinical translation of breast cancer treatment.

## Materials and methods

### Materials

Poly (lactic-co-glycolic acid)-carboxylic acid (PLGA-COOH) with a molecular weight of 20,000 Da (LA:GA = 50/50) was obtained from Jinan Daigang Biotechnology Co., Ltd. (Jinan, China). The MMP-2 substrate peptide (EGPLGVRGK) and PEG_3000_-COOH were custom synthesized by Qiyue Biotechnology Co., Ltd. (Xi’an, China). FITC-labeled MMP-2-PEG was also custom synthesized. DSF was obtained from Aladdin Reagent Co., Ltd. (Shanghai, China). Recombinant mouse MMP-2 was purchased from Abcam Inc. (Abcam, Cambridge, Britain), and *p*-aminophenyl mercuric acid (APMA) was obtained from Genmed Scientifics, Inc., USA.

### Synthesis of PFP@PDM-PEG and PFP@PD

To synthesize the PEGylated MMP-2 peptide (EGPLGVRGK-PEG), we first used Fmoc solid-phase peptide synthesis (SPPS) following a known method to create the MMP-2 peptide (EGPLGVRGK). Next, PEG_3000_-COOH was introduced, and the reaction vessel was sequentially washed with dimethylformamide (DMF), dichloromethane (DCM), and methanol before drying. The mixture was then cleaved using a combination of trifluoroacetic acid (TFA), water, *1,2*-ethyldimercaptan (EDT), and triisopropyl silane (TIS), followed by sequential evaporation to eliminate the organic solvent DCM. Finally, the crude product was subjected to dialysis against distilled water (MWCO 3,500 Da) for purification. Then, the PEGylated MMP-2 peptide was affixed to the surface of the PLGA-COOH shell using the standard carbodiimide method, resulting in the formation of PLGA-MMP-2-PEG. This material was subsequently subjected to freeze-drying for further use.

For the next stage, a mixture comprising PLGA-MMP-2-PEG, DSF, and PFP was dissolved in dimethyl sulfoxide (DMSO) and subjected to ultrasonication in an ice bath for 3 min (100 W, on:off = 1:1) using a sonicator (Sonics & Materials Inc., Newtown, CT, USA). Next, 8 ml of a polyvinyl alcohol solution (4% w/v) was introduced, and the ultrasonication process was repeated under the same conditions. Subsequently, a solution of isopropyl alcohol (10 ml, 2% v/v) was added, and the mixture was magnetically stirred in an ice bath for 3 h to remove the organic solvent. After this process, the PFP@PDM-PEG nanodroplets were obtained by centrifugation (4 °C, 12,000 rpm, 8 min), followed by washing and resuspension.

To optimize the synthesis of the nanodroplets, we tested different feeding ratios of DSF and PLGA-MMP-2-PEG, including DSF/PLGA-MMP-2-PEG (w/w) ratios of 1:2.5, 1:7.5, 1:12.5, 1:17.5, and 1:22.5. Notably, the feeding ratio of 1:12.5 (w/w) yielded the highest encapsulation efficiency (EE) (Additional file 1: Fig. S1). Consequently, we utilized this specific feeding ratio in subsequent experiments.

The release curve of DSF was assessed using the dialysis membranes diffusion technique. First, PFP@PDM-PEG solution was equivalently poured into two dialysis membranes (MW cut-off = 2000 Da, n = 3), one of which was irradiated with LIFU (2 W cm^−2^ for 3 min), and the other one was without any treatment. Then, the dialysis membrane was placed into an 80 ml buffer solution containing Tween-80 (v/v = 0.1%) and ethanol (v/v = 30%). Next shake slowly on a shaker (120 rpm, 37 °C). At specific times (0.5, 1, 2, 4, 8, 16, 24, 48 h), 500 μL buffer solution was collected for measurement by the HPLC system and replaced by 500 μL of a fresh buffer solution. Finally, the cumulative release ratio of DSF in the two groups was calculated.

For the synthesis of the PFP@PD nanodroplets, a mixture containing PLGA-COOH (50 mg), DSF (5 mg), and PFP (200 µl) was dissolved in DMSO and subjected to ultrasonication. The subsequent steps mirrored the procedure described above. Additionally, fluorescent dyes, such as DiI, DiR or FITC, were introduced during the synthesis process to confer the nanodroplets with fluorescence imaging capability.

### Characterization of the PFP@PDM-PEG and PFP@PD nanodroplets

The morphology of the PFP@PDM-PEG and PFP@PD nanodroplets was examined using transmission electron microscopy (TEM, Hitachi H-7500, Tokyo, Japan) and scanning electron microscopy (SEM, Hitachi SU8010, Tokyo, Japan). Particle size and zeta potential were determined using a Malvern Zetasizer Nano ZS90 (UK). Nuclear magnetic resonance (^1^H NMR) spectra were recorded on an NMR spectrometer (Bruker AVANCE NEO 400, Bruker BioSpin GmbH, Rheinstetten, Germany) to confirm the successful formation of covalent bonds between PLGA-COOH, the MMP-2 substrate peptide, and PEG.

To quantitatively evaluate the binding rate of FITC-labeled MMP-2-PEG to PLGA-COOH, flow cytometry (FCM) was employed (Sonic SH800, Japan), using PFP@PD nanodroplets as a control. The excitation wavelength was set to 488 nm.

### Cell culture and animal model

The 4T1 cell line (a mouse TNBC cell line) was maintained in RPMI-1640 medium supplemented with 10% fetal bovine serum (FBS, v/v) and 1% penicillin‒streptomycin (v/v) under standard conditions (5% CO_2_, 37 °C). All experiments involving mice were conducted following the approval of the Animal Care and Use Committee of Chongqing Medical University. Female BALB/c mice (6–8 weeks old) were procured from Chengdu Dossy Experimental Animals Co., Ltd. The 4T1 tumor-bearing model was established by subcutaneously injecting 1 × 10^7^ 4T1 cells into the right lower abdomen of each mouse.

### In vitro cellular uptake and in vivo biodistribution

#### In vitro cellular uptake

In the in vitro cellular uptake study, 4T1 cells were seeded in 3.5 cm confocal dishes at a density of 2 × 10^4^ cells per dish. DiI-labeled PFP@PDM-PEG (with an equal PLGA concentration of 1.0 mg mL^−1^) was added for incubation at 37 °C for 1 h in the absence or presence of APMA-activated MMP-2 (1 µg mL^−1^). Subsequently, the pretreated nanodroplets were added to each well for 1, 2, or 4 h of coincubation. After a 20-min incubation with DAPI, the cells were examined using confocal laser scanning microscopy (CLSM, OLYMPUS confocal microscope CSU-W1, Japan). Additionally, flow cytometry (FCM, Sonic SH800, Japan) was employed to assess the impact of MMP-2-triggered PEG deshielding on 4T1 cell uptake.

#### In vivo biodistribution

For the in vivo biodistribution experiment, tumor-bearing mice were randomly divided into two groups (n = 3): the PFP@PDM-PEG group and the PFP@PD group. Each group of mice received an intravenous injection of DIR-labeled nanodroplets at a dose of 10 mg kg^−1^ DSF. Small animal fluorescence imaging equipment (AniView 600 pro, Guangzhou, China) was used to monitor the accumulation of nanodroplets in the tumor region at various time points (0, 3, 6, 12, 24, and 36 h after injection). Additionally, three mice from each group were euthanized after 24 h, and their major organs (heart, liver, spleen, lungs, kidneys) and tumor tissues were collected for fluorescence imaging. Quantitative analysis was performed on the obtained fluorescence images.

### Drug penetration

To evaluate the ability of the drug delivering nanodroplets to penetrate from the surface to the core, 3D spheroid models of 4T1 cells were constructed. Using 6-well ultralow adherent plates, 1 × 10^6^ 4T1 cells were cultured for 10 days. The cells were then incubated with FITC-labeled PFP@PDM-PEG (with an equal PLGA concentration of 1.0 mg mL^−1^) at 37 °C for 1 h in the absence or presence of APMA-activated MMP-2 (1 µg mL^−1^). Subsequently, pretreated nanodroplets were added to 3D cell spheroids with or without LIFU irradiation. After 4 h of coincubation, CLSM was used for observation. Scanning various layers from the surface to the interior of the tumor spheroids was conducted to quantify the penetration depth. For the assessment of intratumoral drug penetration, mice bearing 4T1 tumors were randomly divided into three groups: the PFP@PD group, the PFP@PDM-PEG group, and the PFP@PDM-PEG + LIFU group. In the PFP@PDM-PEG + LIFU group, a LIFU device (LMSC051 ACA; Institute of Ultrasound Imaging, Chongqing Medical University, Chongqing, China) was employed. The nanodroplets were labeled with the fluorescent dye DiI. Ultrasound irradiation was administered 24 h after injection. Confocal laser scanning microscopy (CLSM) was performed on frozen tumor slices to directly observe drug penetration after staining with DAPI.

### In vitro and in vivo ultrasound imaging (USI) performance

#### In vitro phase transition confirmation

To confirm the acoustically induced phase transition capability of the PFP@PDM-PEG, the nanodroplets were excited by LIFU, and their phase transition was observed under an optical microscope. In Vitro Ultrasound Imaging (USI) Performance: The in vitro ultrasound imaging (USI) performance of PFP@PDM-PEG was assessed by diluting a sample of PFP@PDM-PEG and placing it in a perforated 3% agarose gel phantom. The sample was then exposed to pulsed-wave LIFU irradiation for durations ranging from one to four minutes at power levels of 1–4 W cm^−2^. Ultrasound images were captured before and after each LIFU treatment to determine the most suitable LIFU parameters for in vivo evaluation.

#### In vivo study

4T1 tumor-bearing mice were randomly divided into two groups. Each group received an intravenous injection of PFP@PD or PFP@PDM-PEG (200 μL, with an equal PLGA concentration of 1.0 mg mL^−1^). After 24 h, US mode, contrast-enhanced ultrasound (CEUS) mode, and peak intensity (PI) mode images were acquired at the tumor site both before and after LIFU treatment (2 W cm^−2^, 3 min). The analysis of ultrasonic intensity was conducted using DFY ultrasound imaging analysis software developed by the Institution of Ultrasound Imaging at Chongqing Medical University, China.

### Antitumor efficacy of PFP@PDM-PEG in vitro*.*

#### In vitro cytotoxicity evaluation

4T1 cells were seeded in 96-well plates at a density of 4 × 10^3^ cells per well and incubated for 24 h. Subsequently, the cells were cultured for an additional 24 h in fresh medium (2% FBS) containing varying concentrations of DSF or PFP@PDM-PEG (equivalent DSF concentrations of 250 nM, 500 nM, 750 nM, 1000 nM, 1500 nM, and 2000 nM). The PFP@PDM-PEG group was further divided into two subgroups, with or without APMA-activated MMP-2 pretreatment (1 µg mL^−1^). After a 2-h incubation with 10 μL of CCK-8 reagent, the viabilities of the cells in each well were determined using a microplate reader (Cytation 3, BioTek, Vermont, USA) at a wavelength of 450 nm. The cell viability at the half maximal inhibitory concentration (IC50) was calculated using the provided equation considering the background absorbance of the medium.

#### Cell apoptosis assay

4T1 cells were seeded into a six-well plate and cultivated for 24 h. Subsequently, 4T1 cells were randomly assigned to different groups (n = 3): (i) Control group, (ii) LIFU group (exposed only to LIFU), (iii) DSF group, (iv) PFP@PDM-PEG group, (v) PFP@PDM-PEG with APMA-activated MMP-2 (1 µg mL^−1^), and (vi) PFP@PDM-PEG with APMA-activated MMP-2 (1 µg mL^−1^) + LIFU group (LIFU irradiation was applied after a 4-h incubation. After 24 h, all cells were collected, stained with Annexin V-FITC/PI apoptosis detection kit, and analyzed for cellular apoptosis using FCM.

### Assessment of cell migration and invasion

Cell Migration: 4T1 cells (2.5 × 10^5^ per well) were initially seeded in 6-well plates. When the cell density reached 80–90%, a precise denuded area was created in the center of each well using a 10 μl pipette tip. Subsequently, the detached cells were washed away with PBS. The cells were then subjected to various treatments, including serum-free medium, DSF, and PFP@PDM-PEG (with an equivalent DSF concentration of 1000 nM). The PFP@PDM-PEG group was further subdivided into two groups for pretreatment with APMA-activated MMP-2 (1 µg mL^−1^) or not. To exclude any potential effects of LIFU on cell migration, all groups received LIFU irradiation (2 W cm^−2^, 3 min) after a 4-h incubation. After 24 h, microscopic observations were made, image collection was performed, and the scratch width was quantitatively analyzed using ImageJ software (version 1.52 g, Java 1.80_172, NIH).

Cell Invasion: For the cell invasion study, 1 × 10^5^ cells were plated on Matrigel-coated Transwell chambers (Costar, Corning, NY, USA). Following the same treatment conditions as described above, 4T1 cells were treated with serum-free medium, DSF, or PFP@PDM-PEG for 24 h. Cells that had migrated to the lower surface of the membrane were fixed with 75% ethanol at 4 °C for 20 min. Then, the cells were stained for 15 min with hematoxylin. Subsequently, the cells in random objective fields were observed at × 10 magnification after being washed three times.

### VM formation assay

For the VM formation assay, an 8-well culture slide (Falcon®, Corning, USA) was coated with 30 μL of Matrigel (Corning Cat. No. 354234) using a thin gel coating method at 37 °C for 15 min. The cells were suspended in serum-free Opti-MEM (Life Technologies, USA) supplemented with 1% GlutaMAXTM (Life Technologies, USA) and placed on the Matrigel-coated surface. Each well received 2.5 × 10^5^ cells and was subjected to various treatments based on the experimental group (such as those in the cell migration study). The cells were then incubated for 24 h. After incubation, the slides were examined and photographed using an inverted microscope (OLYMPUS, Tokyo, Japan). Quantitative analysis of VM formation was performed using ImageJ software.

### In vivo VM targeted therapy

To evaluate the antitumor efficacy of PFP@PDM-PEG due to its inhibition of VM formation in vivo, 4T1 tumor-bearing mice were randomly assigned to different groups (n = 5):

(i) Control group, (ii) LIFU group (exposed only to LIFU), (iii) DSF group, (iv) PFP@PD group, (v) PFP@PDM-PEG group, and (vi) PFP@PDM-PEG + LIFU group. Mice were administered 200 μL of normal saline, PFP@PD, PFP@PDM-PEG, or DSF suspension via the tail vein (equivalent DSF concentration: 10 mg kg^−1^). LIFU irradiation (2 W cm^−2^, 3 min) was applied to the tumor area 24 h postinjection. Tumor dimensions were measured every two days, and tumor volume was calculated using the formula (length × width^2)/2 mm^3^. After two weeks, blood samples were collected from the orbit to assess routine blood indices and biochemical indices. Subsequently, the mice in each group were euthanized, and major organs (heart, liver, spleen, lungs, and kidneys) were stained with H&E to evaluate histopathological toxicity. The metastatic burden in both the lungs and liver was evaluated by counting the number of metastatic nodules in the histopathological whole-slide images (WSIs). After staining with CD34/PAS (Abcam, ab8536, Cambridge, MA), COL1 (Affinity Biosciences, AF7001, Jiangsu, China), or activated MMP-2 (Abcam, ab92536, Cambridge, MA), the tumor tissues were examined to determine the mechanism by which PFP@PDM-PEG inhibits VM development. Furthermore, the protein levels of COL1 and active MMP-2 in tumor tissues were quantified through Western blot analysis.

### Statistical analysis

The results are presented as the mean ± standard deviation of the mean for each sample. Statistical analysis was performed using one-way analysis of variance (ANOVA) and t tests in GraphPad Prism 9.0 software (GraphPad Prism software, San Diego, CA, USA) to compare data between different groups.

## Results and discussion

### Characterization of PFP@PDM-PEG

**Fig. 1:**
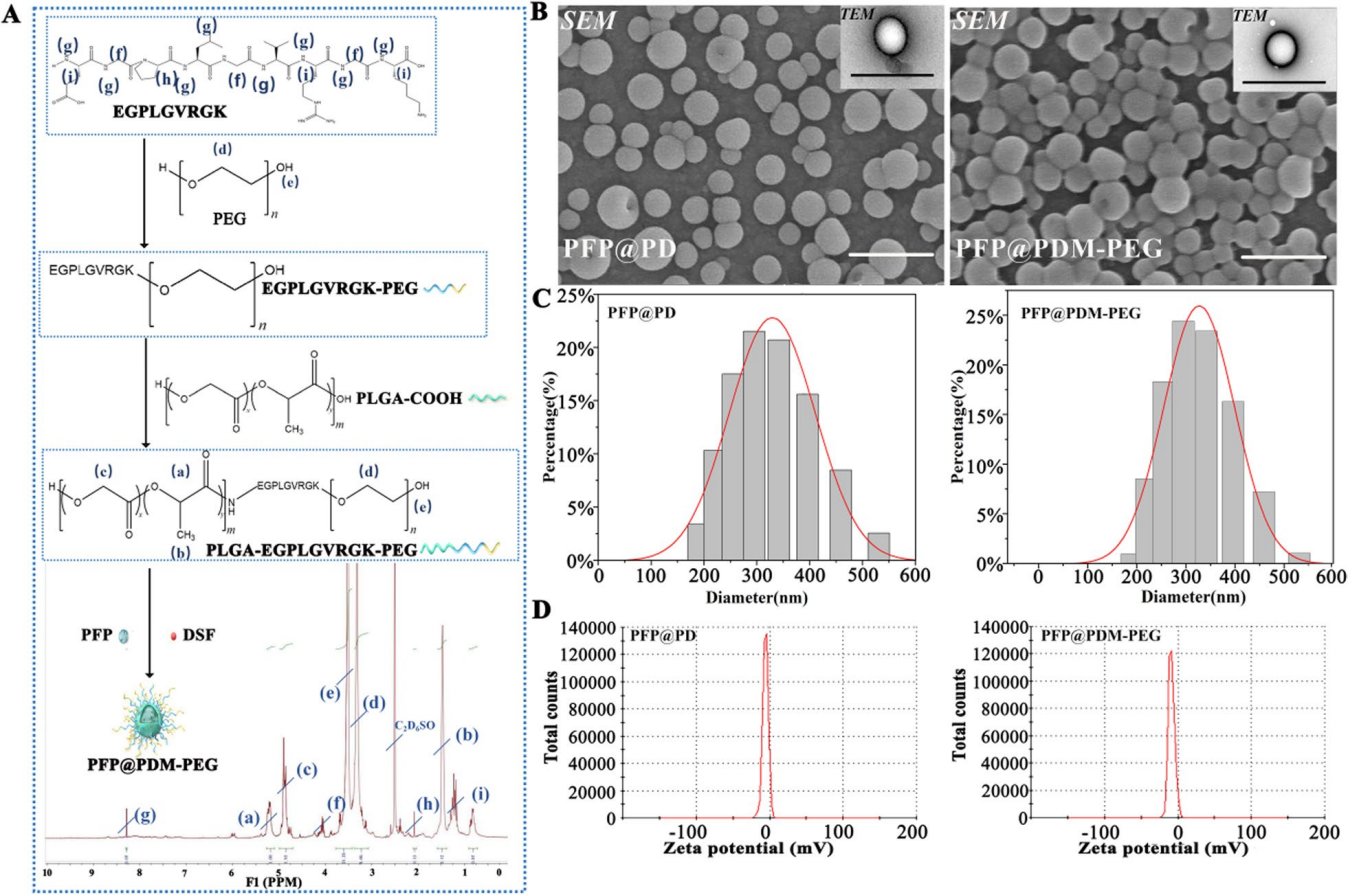
Characterization of PFP@PD and PFP@PDM-PEG.** A** Composite diagram and ^1^H NMR analysis of PFP@PDM-PEG. **B** SEM and TEM images of PFP@PD and PFP@PDM-PEG; scale bar: 500 nm. **(C)** The particle sizes of PFP@PD and PFP@PDM-PEG. **D** The zeta potential distributions of PFP@PD and PFP@PDM-PEG

As shown in Fig. 1A, ^1^H NMR spectroscopy (200 MHz, [D8] THF, 25 °C, TMS) was performed to verify the successful conjugation of PLGA-COOH and MMP-2-PEG. Peaks corresponding to the free MMP-2 substrate peptide were detected at *δ* 3.9 (H_f_), 8.3 (H_g_), 1.9 (H_h_), and 1.2 (H_i_) ppm. Peaks for PLGA were observed at *δ* 5.2 (H_a_), 1.5 (H_b_), and *δ* 4.8 (H_c_) ppm. Additionally, PEG proton peaks were identified at both *δ* 3.6 (H_e_) and *δ* 3.5 (H_d_) ppm. Furthermore, the binding rate of FITC-labeled MMP-2-PEG to PLGA-COOH was quantitatively evaluated to be approximately 99% (Additional file 1: Fig. S2). These results confirmed the successful and efficient conjugation of PLGA-COOH to MMP-2-PEG, which provided conducive support for further preparation of LIFU/MMP-2 dual-responsive PFP@PDM-PEG. Next, a simple emulsion evaporation method was used to synthesize PFP@PDM-PEG. Transmission electron microscopy (TEM) and scanning electron microscopy (SEM) images of PFP@PDM-PEG were collected to observe the morphology and uniformity of the nanodroplets. The results illustrated that PFP@PDM-PEG and PFP@PD exhibited spherical structures with a uniform dispersity (Fig. 1B). Then, dynamic light scattering (DLS) was utilized to determine the hydrodynamic size and surface zeta potential of the nanodroplets. As depicted in Fig. 1C. The average diameter of PFP@PDM-PEG, measured to be approximately 343 nm, was slightly larger than that of PFP@PD (approximately 305 nm). Furthermore, the surface potential shifted from -5.6 mV to -9.3 mV upon modification with MMP-2 peptide-terminated PEG (Fig. 1D). HPLC was used to detect and establish the standard curve of DSF, and the content of DSF in the nanodroplets was calculated based on this standard curve (Additional file 1: Fig. S3). The encapsulation efficiency (EE) and loading capacity (LC) of DSF in the nanodroplets were calculated using the following methods (n = 3):

$${\text{EE}}=\frac{\mathrm{DSF\; encapsulated\; in\; nanodroplets}}{\mathrm{Total\; DSF\; added}}$$× 100%

$${\text{LC}}=\frac{\mathrm{DSF\; encapsulated\; in\; nanodroplets}}{\mathrm{Weight\; of\; nanodroplets}}$$× 100%

Additional file 1: Table S1 illustrates that the LC of the nanodroplets was 6.8% for PFP@PDM-PEG and 7.1% for PFP@PD. Additionally, the EE was found to be 75.7% for PFP@PDM-PEG and 77.6% for PFP@PD.

The acoustic droplet vaporization (ADV) effect promotes drug release from the nanodroplets into the surrounding medium after microbubble collapse. Approximately 44.23% of the total DSF was released from the PFP@PDM-PEG + LIFU group after 48 h, which was significantly higher than the 6.47% released by the PFP@PDM-PEG without LIFU group (Additional file 1: Fig. S4). These results demonstrated that PFP@PDM-PEG has excellent stability with little drug leakage in the bloodstream. After LIFU irradiation, the drug offloading in the tumor was accelerated.

### Antitumor efficacy of PFP@PDM-PEG in vitro.

#### In vitro cytotoxicity of PFP@PDM-PEG

The cytotoxicity of free DSF, PFP@PDM-PEG without MMP-2, and PFP@PDM-PEG with MMP-2 (nanodroplets pretreated with 1 µg mL^−1^ activated MMP-2) was evaluated using the CCK-8 assay (Additional file 1: Fig. S5A). No significant cytotoxicity was observed at DSF concentrations below 750 nM. However, when the DSF concentration reached or exceeded 1000 nM, the groups with encapsulated DSF showed lower cell viability rates than did the free DSF group, indicating that the affinity of hydrophobic DSF for cancer cells had improved [40]. The encapsulated DSF exhibited increased cytotoxicity, likely due to improved cellular uptake [41]. In particular, compared with the same concentrations of free DSF and nanodroplets without MMP-2 pretreatment, the cells treated with MMP-2-pretreated nanodroplets had significantly lower viability rates. This can be attributed to responsive cleavage of the MMP-2 substrate peptide and enhanced endocytosis of the nanodroplets via dePEGylation. The IC50 values of 4T1 cells treated with DSF, PFP@PDM-PEG without MMP-2, and PFP@PDM-PEG with MMP-2 were 2250, 1702, and 1213 nM, respectively (Additional file 1: Fig. S5B). Finally, a DSF concentration of 1000 nM was selected as the minimum effective concentration for subsequent investigations.

#### In vitro anti-tumor therapy

The combination of MMP-2 responsiveness and LIFU irradiation synergistically enhances the apoptotic effect (Additional file 1: Fig. S5C-D). The therapeutic effect of free DSF was limited due to its low solubility in the medium. However, the delivery efficiency of DSF was increased after inclusion in the nanodroplets, especially after pretreatment with activated-MMP-2, the apoptosis of cancer cells was increased, which may be attributed to the significantly enhanced uptake of the nanodroplets by cancer cells after MMP-2-induced PEG shedding. After LIFU irradiation, DSF was released more and faster, and the therapeutic effect was the best. The total apoptotic number was about 52.13%.

### In vitro cellular uptake and in vivo biodistribution

**Fig. 2:**
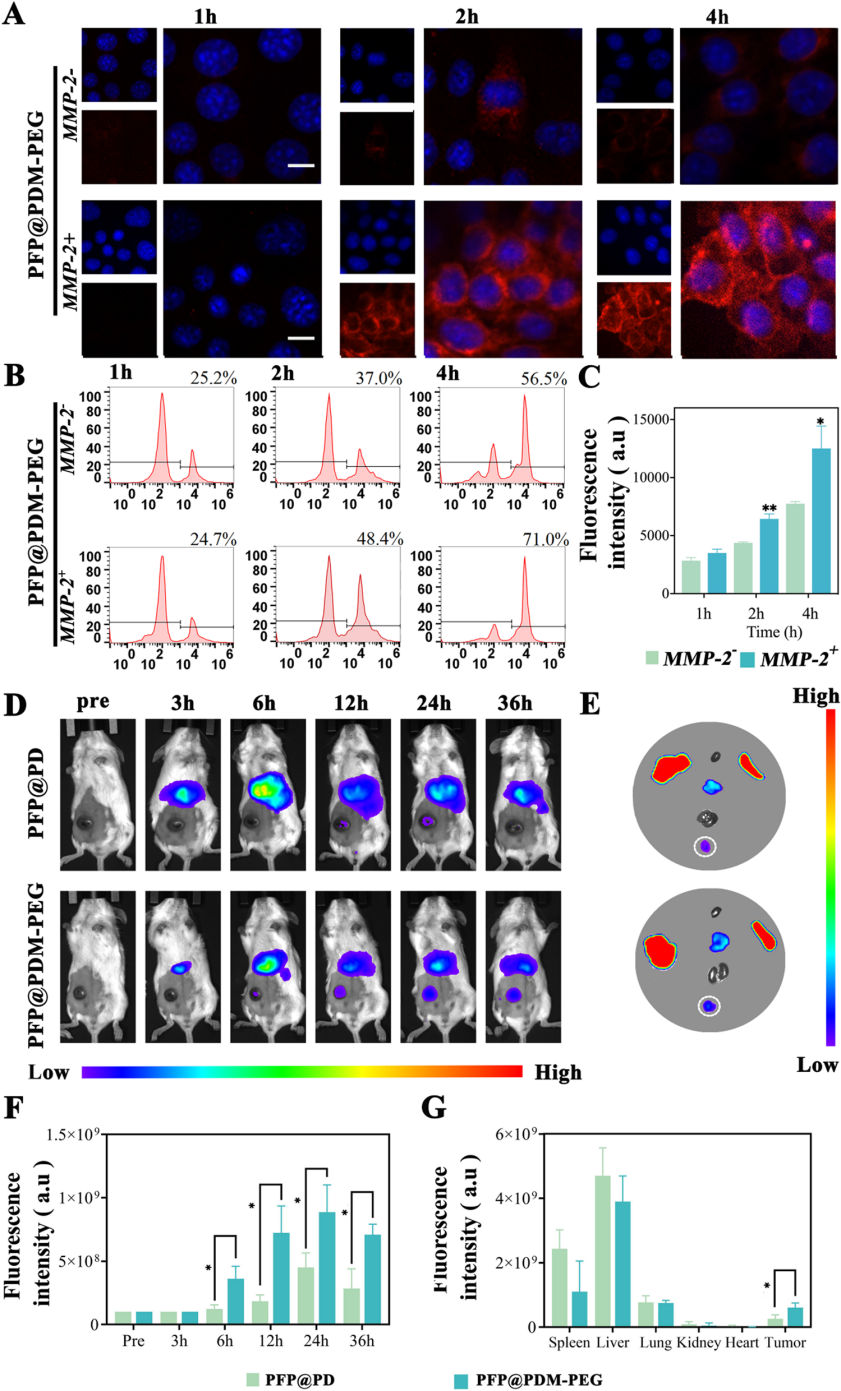
Cellular uptake in vitro and biodistribution in vivo.** A** Confocal laser scanning microscopy (CLSM) images, **B** flow cytometry profiles, and **C** the mean fluorescence intensity, as determined from the CLSM images, of 4T1 cells incubated with MMP-2-treated or MMP-2-free PFP@PDM-PEG at various time points; scale bar: 20 μm (n = 5, t test, *p < 0.05). **D** In vivo fluorescence images of tumor-bearing mice at various time points postinjection and **E** ex vivo fluorescence images of the main organs: heart, liver, spleen, lung, kidney, and tumor (white dotted circles). **F** Quantitative analysis of the fluorescence intensity in the tumor sites at different time points in vivo and **G** quantitative analysis of the corresponding fluorescence intensities of the tissues in vitro (n = 3, t test, *p < 0.05)

To mimic the cellular uptake behavior of the nanodroplets in the tumor microenvironment, specifically in the presence of MMP-2, DiI-loaded nanodroplets were pretreated with activated MMP-2 (1 µg mL^−1^) and subsequently cocultured with 4T1 cells. The fluorescence intensity of the DiI-labeled nanodroplets (red) around the cell nuclei (blue) increased over time. Importantly, the MMP-2-pretreated group exhibited significantly stronger red fluorescence, indicating more efficient cellular internalization **(**Fig. 2A, C). Flow cytometry analysis verified these observations, showing a 1.3-fold increase in uptake in the MMP-2-treated group compared to the MMP-2-free group (Fig. 2B).

In vivo, the tumor-bearing mice were divided into the PFP@PDM-PEG and PFP@PD groups. The fluorescent dye DiR was encapsulated in the nanodroplets for real-time monitoring. Optical imaging using an in vivo fluorescence imaging system was performed at various time points after intravenous injection. The PFP@PDM-PEG group displayed a gradual increase in the fluorescence signal that peaked at 24 h, signifying superior tumor accumulation. In contrast, the PFP@PD group exhibited minimal fluorescence signals at the tumor site, which were significantly lower than those observed in the PFP@PDM-PEG group (Fig. 2D–F). Quantitative analysis of the tissues in vitro (heart, liver, spleen, lungs, kidneys, and tumor) at the time when the tumor fluorescence was the strongest confirmed these results (Fig. 2G). In summary, PFP@PDM-PEG, modified with the MMP-2-PEG peptide, exhibited enhanced tumor accumulation that was attributed to PEGylation-enhanced EPR effect-mediated "passive" tumor targeting and MMP-2-induced dePEGylation in the tumor microenvironment.

PEGylation improves stability and circulation time by creating a hydrophilic and negatively charged coating on nanoparticles, but it also hinders cellular uptake [35]. To overcome this limitation, PEG molecules are incorporated onto nanoparticle surfaces for responsive de-PEGylation, allowing adaptation to the tumor or intracellular environment. MMP-2, an enzyme overexpressed in malignant cancers, has been investigated as a trigger for de-PEGylation [42]. By cleaving peptide linkers in the tumor environment, PEGylated nanodroplets can simultaneously remove their PEG layers where MMP-2 is overexpressed, enhancing selective intracellular uptake [42, 43]. This study also highlights the promising PEG detachment mechanism of the designed PFP@PDM-PEG, which facilitates the cellular uptake of nanodroplets.

### Deep penetration of the LIFU/MMP-2 dual-responsive PFP@PDM-PEG

**Fig. 3:**
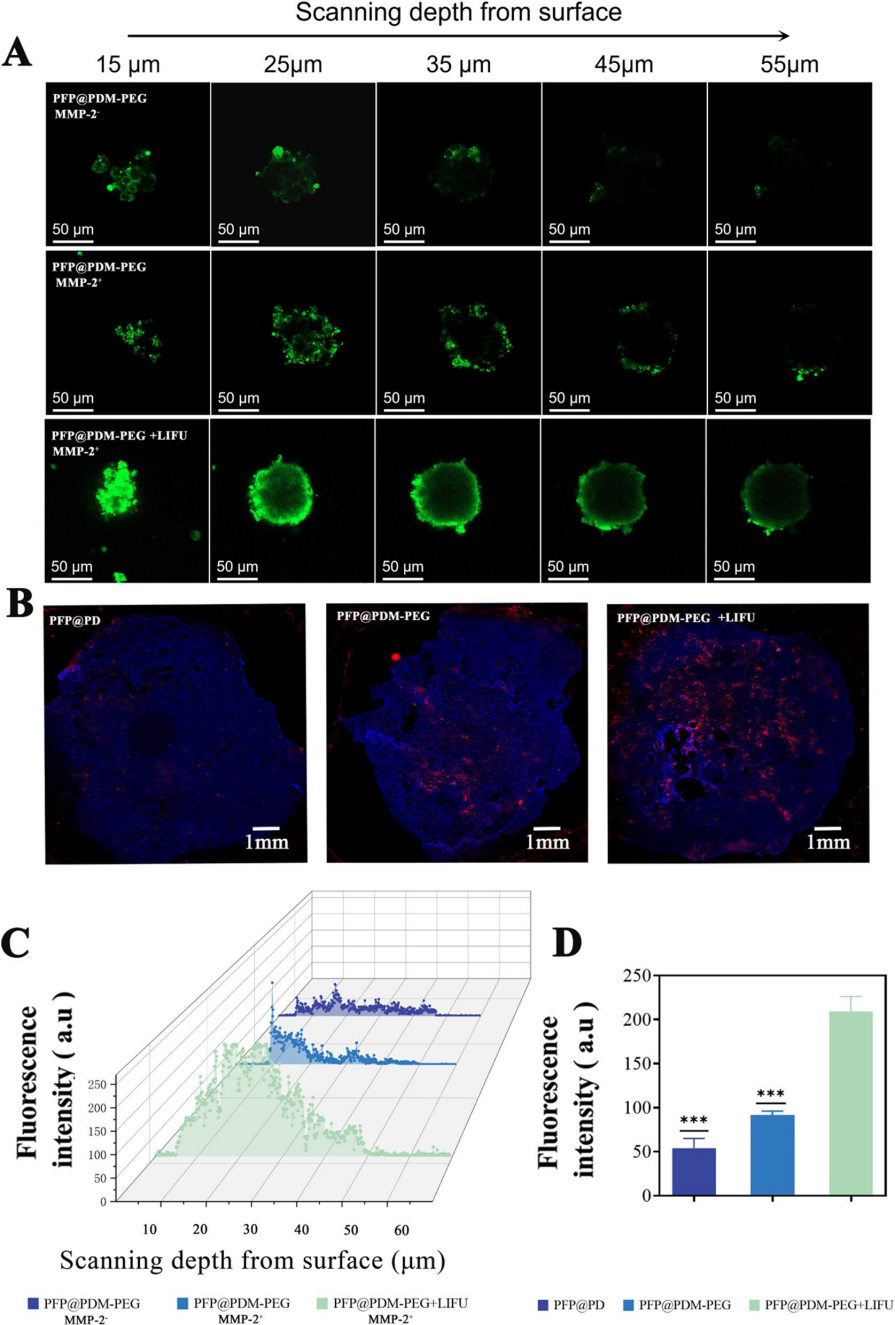
Penetration of drugs delivered by PFP@PDM-PEG into 4T1 tumor spheroids and the tumor parenchyma. **A** In vitro confocal microscopy images of the penetration of FITC-labeled PFP@PDM-PEG into a 4T1 tumor spheroid model and **C** the corresponding fluorescence intensities. Images were obtained from the top to the middle of the spheroids every 10 mm; scale bar: 50 μm. **B** CLSM images of the distribution of DiI-labeled PFP@PDM-PEG in tumor cryosections and **(D)** the corresponding fluorescence intensities; scale bar: 1 mm (n = 3, t-test, ***p < 0.001)

To evaluate the ability the LIFU/MMP-2 dual-responsive PFP@PDM-PEG nanodroplets to penetrate deeply into tumors, a three-dimensional 4T1 cell tumor spheroid was established to mimic the high cell density and increased interstitial pressure characteristic of tumors. After coincubation with FITC-labeled nanodroplets for 4 h, a significant increase in green fluorescence was observed in the de-PEGylated group (PFP@PDM-PEG MMP-2^+^) compared to the PEGylated group (PFP@PDM-PEG MMP-2^−^), confirming the enhanced uptake of drugs or nanoparticles that occurred after MMP-2-responsive dePEGylation. However, the fluorescence was mainly concentrated at the periphery of the tumor spheroid. Following LIFU irradiation, widespread green fluorescence was observed throughout the entire spheroid, penetrating deep into the tumor core (Fig. 3A, C). Analysis of the CLSM images confirmed intratumoral penetration. Compared to the PFP@PDM-PEG group, the PFP@PDM-PEG + LIFU group exhibited a significant increase in fluorescence, with a noticeable increase in DiI-labeled red fluorescence throughout the entire tumor (Fig. 3B, D). These findings demonstrate that the LIFU/MMP-2 dual-responsive delivery system, PFP@PDM-PEG, achieves superior deep penetration to enhance the anti-VM effect of DSF. Additionally, UTMD effectively dilates the vascular endothelial space, which is particularly advantageous in the tumor environment [44–47]. Combining nanodroplets with LIFU enables efficient drug delivery through the dilated space, leading to enhanced drug release and improved therapeutic outcomes. Moreover, UTMD has been shown in the literature to promote penetration. Finally, the MMP-2 response further promotes the uptake of drugs or nanoparticles by deep tumor cells.

### In vitro and in vivo ultrasound imaging

**Fig. 4:**
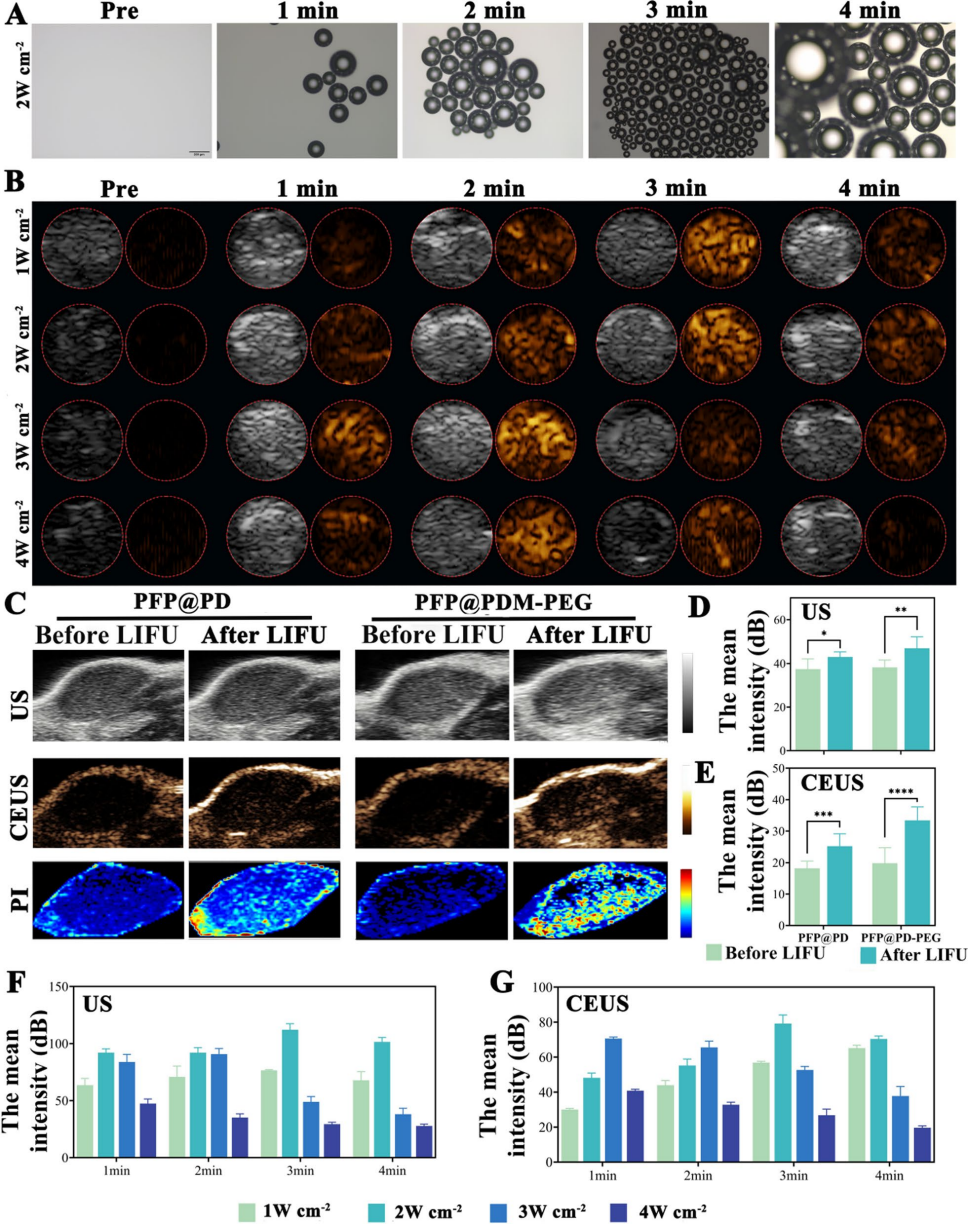
Low-intensity focused ultrasound (LIFU)-driven phase transition and PFP@PDM-PEG ultrasound imaging. **A** Optical microscopy images of PFP@PDM-PEG irradiated with LIFU (2 W cm^−2^) for 0–4 min. Scale bar: 200 μm. **B** Ultrasound images of PFP@PDM-PEG after LIFU irradiation with various parameters. **C** In vivo ultrasound images of US mode, CEUS mode and the parametric peak intensity (PI) color map under LIFU irradiation. Quantitative analysis of the mean intensity in the corresponding US mode **D** and CEUS mode **E**. Quantitative analysis of the mean intensity in B mode **F** and CEUS mode **G** at different time points (n = 3, t test, *p < 0.05, **p < 0.01, ***p < 0.001, ****p < 0.0001)

When exposed to LIFU irradiation, the PFP@PDM-PEG nanodroplets undergo acoustic droplet evaporation, when enables ultrasound imaging. First, optical microscopy was used to observe the liquid‒gas phase transition of PFP within PFP@PDM-PEG. After LIFU irradiation (2 W cm^−2^, 3 min), the size of the PFP@PDM-PEG increased from nanometers to micrometers. The number of PFP@PDM-PEG particles that had undergone phase change increased with increasing excitation time **(**Fig. 4A). Next, the in vitro ultrasound imaging effects of PFP@PDM-PEG with different LIFU parameters, both in B mode and contrast-enhanced ultrasound (CEUS) mode, were observed. As shown in Fig. 4B, the optimal US imaging effect of PFP@PDM-PEG was achieved by performing LIFU (2 W cm^−2^) for 3 min. Quantitative analysis of the echo intensity supported this finding, as the peak value was reached under these conditions, which was consistent with the CEUS images (Fig. 4D, E).

For the in vivo US imaging experiment, ultrasound images of the tumor site (with or without LIFU irradiation) were collected 24 h after nanodroplet injection based on the biodistribution results. Immediate ultrasound imaging revealed that the PFP@PDM-PEG group displayed more pronounced CEUS signals than did the PFP@PD group (Fig. 4C). Furthermore, the parametric peak intensity (PI) color map provided additional evidence that MMP-2-responsive de-PEGylation played a role in enhancing the tumor accumulation of PFP@PDM-PEG. The quantitative analysis results corroborated the in vivo ultrasound images, validating these findings (Fig. 4F, G). The results validated the distinguished US imaging capabilities of the PFP@PDM-PEG NPs both in vivo and in vitro, which is conducive to further vasculogenic mimicry inhibition therapy and real-time image monitoring.

### Inhibition of VM formation and cell motility in vitro

**Fig. 5:**
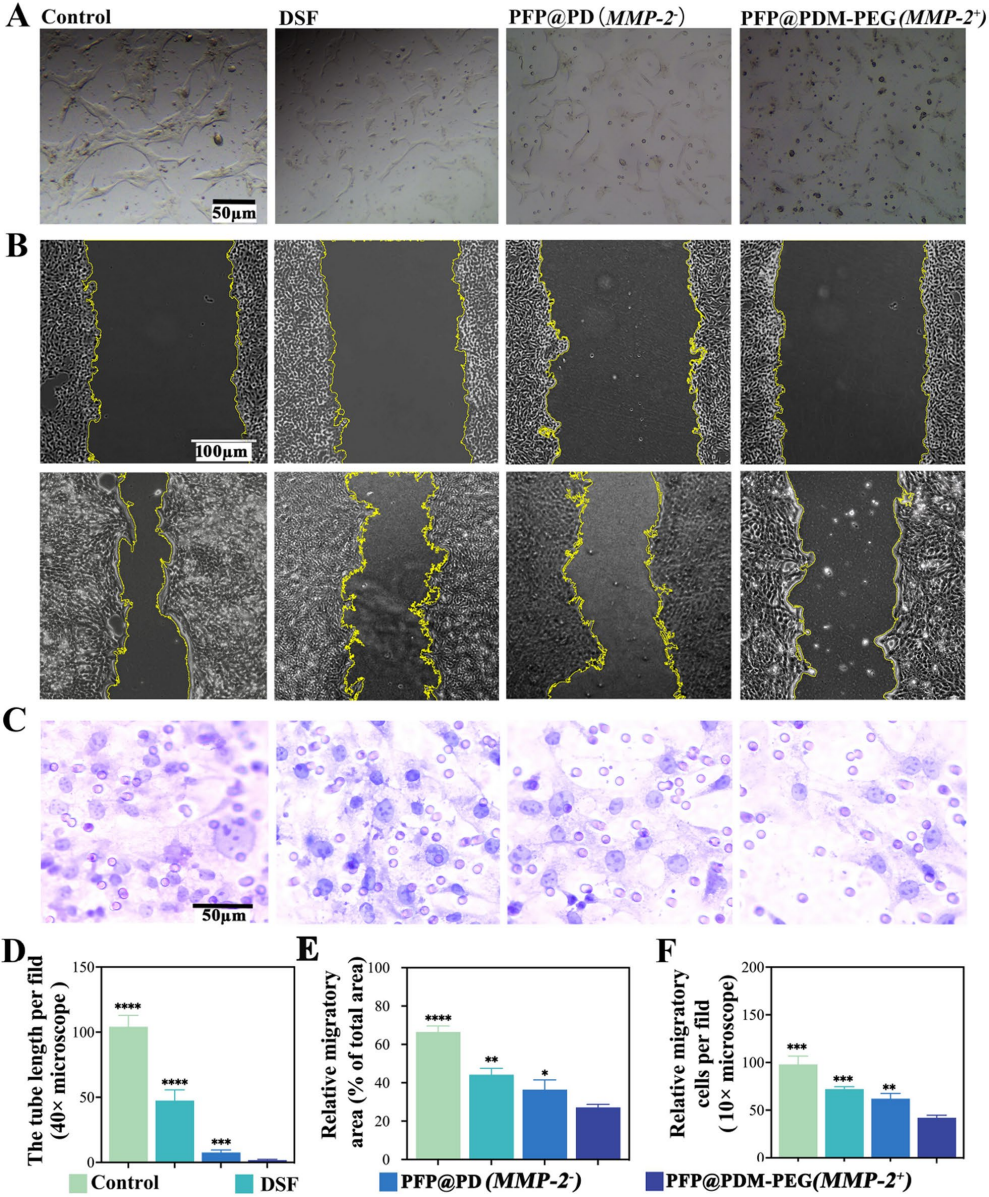
Inhibition of VM formation and cell motility in vitro.** A** Images showing the inhibitory effects of different treatments on tubular formation; scale bar: 50 μm. **B** Representative images of cell migration from the cell scratch assay; scale bar: 100 μm. **C** Representative images of 4T1 cells in the bottom layer of Transwell chambers 24 h after different treatments; scale bar: 50 μm. **D** Semiquantitative analysis of the relative tube length per field. **E** Semiquantitative analysis of the relative migratory area. **F** Semiquantitative analysis of the number of migrating cells per field (10 × magnification) (n = 3, t test, *p < 0.05, **p < 0.01, ***p < 0.001, ****p < 0.0001)

VM can result from tumor cells through mechanisms such as tubular formation, migration, and invasion deformation [17]. Therefore, assessing these behaviors posttreatment is crucial for the development of VM-inhibiting therapies. We evaluated the ability of 4T1 cells to form VM channels in vitro using Matrigel. As shown in Fig. 5A and D, 4T1 cells exhibited some capacity to form capillary-like tubes on Matrigel overnight. However, after treatment with MMP-2-treated PFP@PDM-PEG, the cells formed few typical vasculogenic-like structures (1.8 ± 0.7%), which was significantly less than that in the control group. DSF (46.0 ± 11.3%) and MMP-2-free PFP@PDM-PEG (7.3 ± 1.4%) also reduced tubular formation by cells. Figure 5B and E show the inhibitory effects of free DSF and DSF-loaded PFP@PDM-PEG on 4T1 cell migration as determined by a cell scratch assay. Compared to the control group, the DSF-loaded responsive nanosystem treated with MMP-2 exhibited the most potent blocking effect, resulting in the smallest cell migration area (27.1 ± 1.6%). Similarly, the Transwell invasion assay demonstrated that the MMP-2-treated PFP@PDM-PEG group had significantly fewer invading cells (42.0 ± 2.6) than the other groups (Fig. 5C, F). Thus, this DSF-loaded responsive nanosystem shows promise for effectively inhibiting cell migration and invasion.

This study robustly demonstrated that these nanodroplets can effectively inhibit the tubular formation by and the invasion and migration of tumor cells, indicating strong in vitro potential for VM inhibition and supporting further in vivo applications.

### In vivo antitumor therapy and inhibition of distant metastasis

**Fig. 6:**
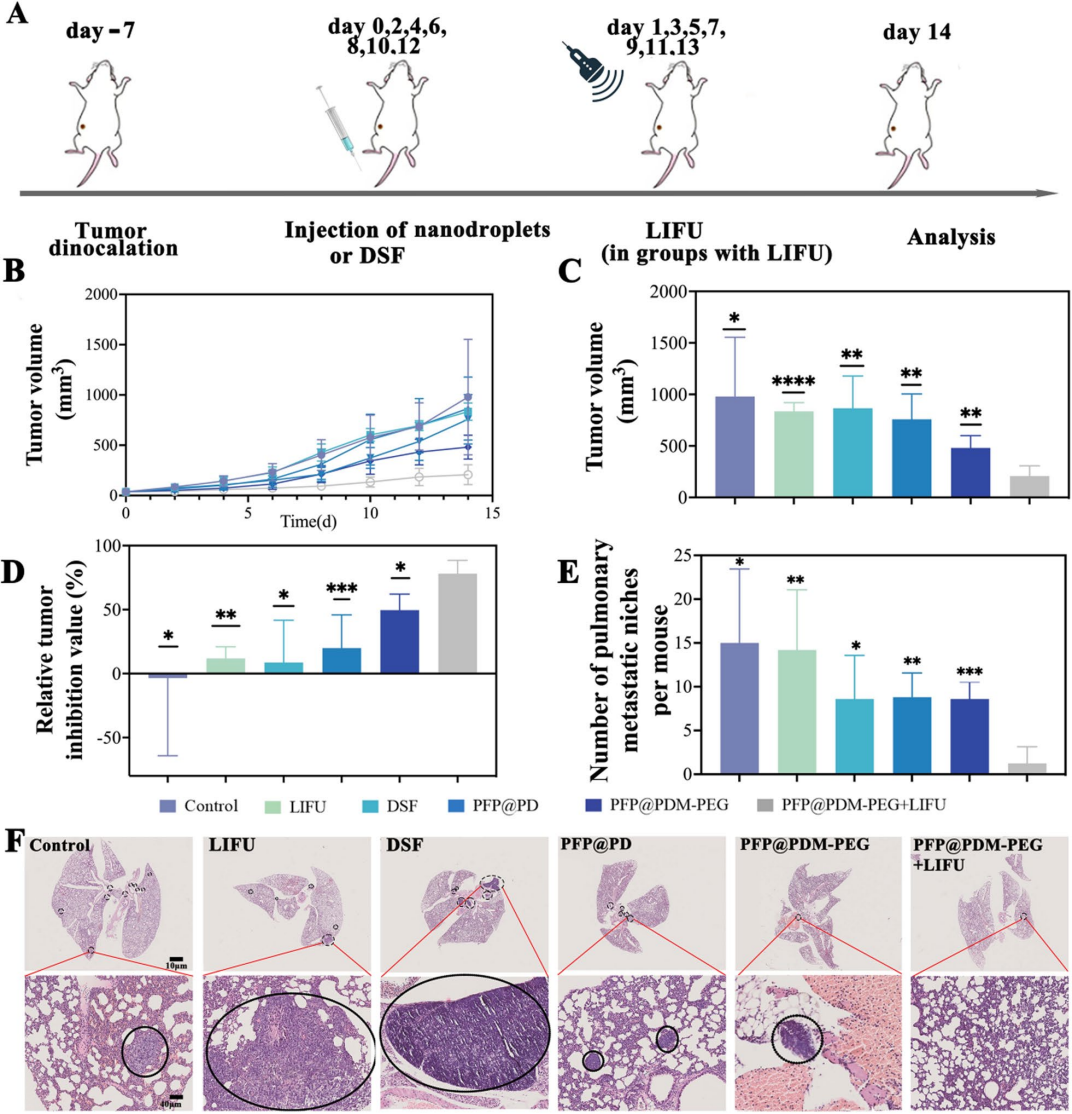
In vivo antitumor therapy and inhibition of distant metastasis. **A** Schematic of the in vivo experimental design. **B** Tumor growth curves. **C** Tumor volumes on Day 14. **D** Tumor growth inhibition values. **E** Number of macroscopically visible pulmonary metastases in the different treatment groups. **F** Representative H&E staining images of lungs with metastatic deposits (black circles) following treatment; scale bars: 10 μm (above) and 40 μm (below) (n = 5, mean ± SD, t test, *p < 0.05, **p < 0.01, ***p < 0.001, ****p < 0.001)

Mouse breast cancer models were constructed to assess the antitumor therapeutic effects of vasculogenic mimicry inhibition with the LIFU/MMP-2 dual-responsive nanodroplets. LIFU irradiation was performed on the mice 24 h after intravenous injection of the nanodroplets. During the observation period of 14 days, a total of 7 treatments were performed (Fig. 6A). The PEG modification and responsiveness from the MMP-2 peptide allowed more PFP@PDM-PEG to accumulate in the tumor area, resulting in smaller tumor volumes in the responsive PFP@PDM-PEG group. Without LIFU excitation, the antitumor effect of PFP@PDM-PEG alone was limited due to the slow and limited release of DSF. During treatment, the tumor growth rate of the mice in the PFP@PDM-PEG + LIFU group was the slowest (Fig. 6B). Similarly, the tumor volume in the PFP@PDM-PEG + LIFU group was the smallest among all the groups (Fig. 6C and Additional file 1: Fig. S6), indicating that the PFP@PDM-PEG + LIFU had the most robust antitumor effect. The quantitative analysis supported these significant differences (Fig. 6D). In particular, the PFP@PDM-PEG + LIFU group exhibited the highest rate of tumor growth inhibition, reaching 78.1 ± 10.5% (Fig. 6D). This may be attributed to the targeted controlled release and enhanced penetration of DSF with the dual-responsive PFP@PDM-PEG nanosystem, which reduced the blood supply to the tumor tissue and eventually achieved the desired antitumor effect by inhibiting VM channel formation.

VM formed by tumor cells lacking robust endothelial cells facilitates metastasis along blood vessels and is a significant factor in breast cancer mortality. Thus, we also evaluated the combined effect of the dual-responsive nanodroplets and LIFU on lung metastasis inhibition (Fig. 6E). According to the statistical analysis (Fig. 6F), there was a substantial number of microscopic pulmonary metastases in both the control and LIFU groups, with an average of 15 ± 8.4 and 14.2 ± 6.9 metastatic deposits per mouse, respectively. In contrast, mice treated with PFP@PD, PFP@PDM-PEG, and DSF for 14 days displayed fewer metastatic deposits, averaging 8.8 ± 2.8, 8.6 ± 1.9, and 8.6 ± 1.7 per mouse, respectively. Remarkably, mice administered PFP@PDM-PEG + LIFU exhibited notably fewer microscopic pulmonary metastases, with only 1 ± 1.7 deposits per mouse. This represents a 60% reduction in lung metastases compared to that in the control group, with 3 out of 5 mice in the PFP@PDM-PEG + LIFU group showing no lung metastasis, indicating the potential of PFP@PDM-PEG + LIFU to inhibit distal tumor spread. Notably, no significant hepatic metastases were detected in any of the groups (Additional file 1: Fig. S7). The results showed that while inhibiting the growth of primary tumors, nanodroplet + LIFU treatment significantly suppressed lung metastasis in mice by inhibiting VM formation, suggesting its promising clinical applications.

### In Vivo Mechanisms of Targeted VM Therapy

**Fig. 7:**
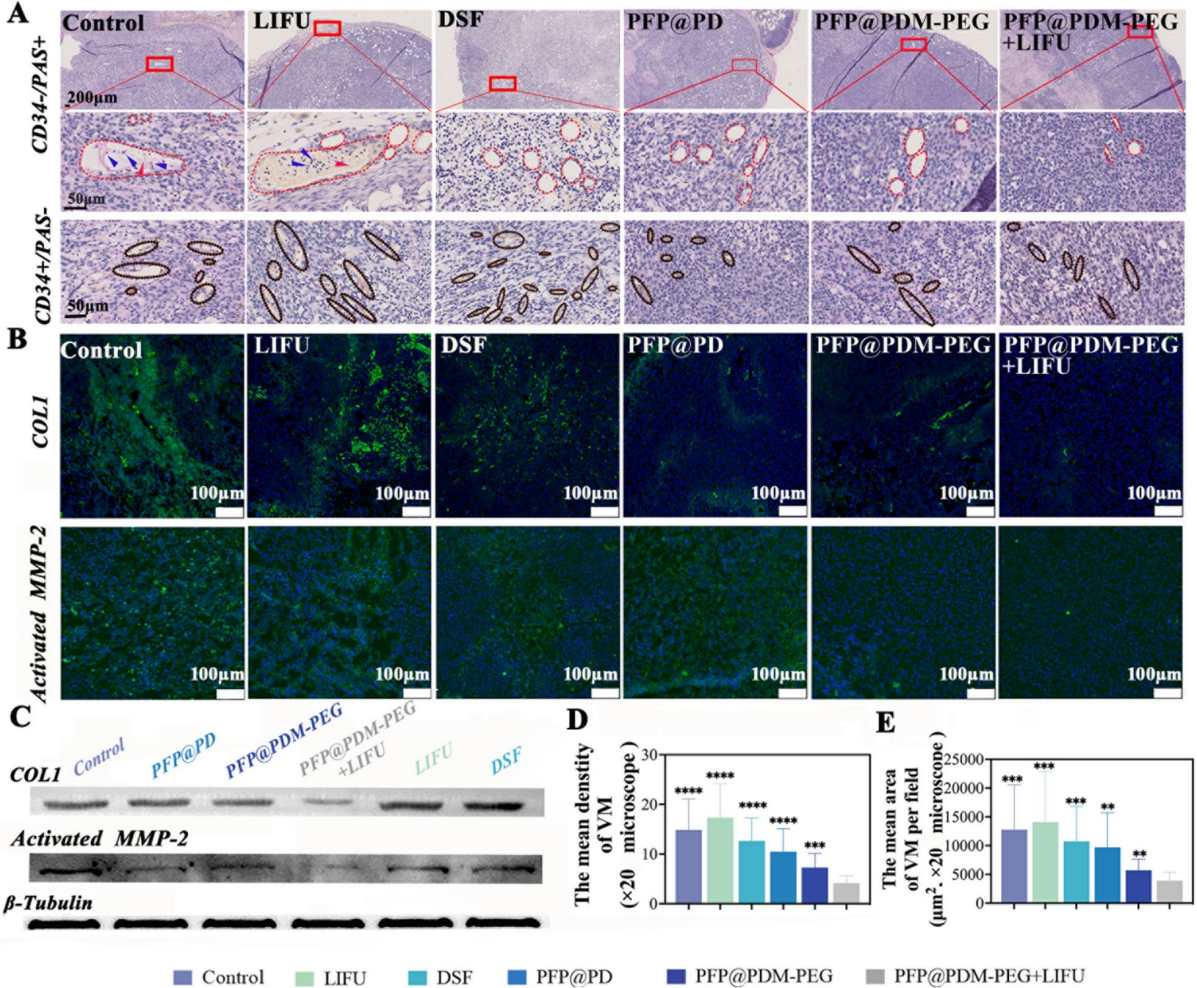
In vivo VM-targeted therapeutic mechanisms.** A** Representative images of VM channels (stained CD34^−^/PAS^+^) and endothelium-dependent microvessels (stained CD34^+^/PAS^−^) from each tumor group. VM channels and endothelium-dependent microvessels are marked in the red and brown dashed areas, respectively; red blood cells are marked with red arrows; and tumor cells in VM channels are marked with blue arrows. **B** Immunofluorescence examination of tumor tissues for COL1 and activated MMP-2 (both shown as green). **C** Western blot analysis of COL1 and activated MMP-2 in tumor tissues. **D** Calculation of the mean density of the VM channels in each group. **E** Analysis of the mean VM area per field (20 × magnification) (n = 3, t test, **p < 0.01, ***p < 0.001, ****p < 0.0001)

To evaluate the impact of treatment on VM channel blockade, we employed the CD34-PAS dual-staining technique, which is considered the gold standard for assessing VM channels in tumor cell-lined VM. In Fig. 7A, the VM channels are shown in red, while the endothelium-dependent microvessels are shown in brown. In the control group, the LIFU group, and the DSF group, VM channel clusters were arranged in circular or oval patterns with larger lumen areas. In contrast, the other three treatment groups had fewer VM structures. Semiquantitative analysis of the microscopic observations revealed that compared with the control treatment, the experimental treatment led to a decrease in the density of VM channels. Notably, the PFP@PDM-PEG + LIFU group exhibited more robust inhibition of VM channels than the other treatment groups (Fig. 7D**, 20** × magnification). Similarly, treatment with PFP@PDM-PEG + LIFU significantly reduced the mean VM area per field in the tumor slices compared to that of the other groups (Fig. 7E**, 20** × magnification). These findings suggest that DSF delivery via PFP@PDM-PEG, with enhanced penetration and precise drug release after LIFU, is superior to free DSF in terms of generating a potent anti-VM effect. Furthermore, red blood cells (indicated by red arrowheads) and shedding tumor cells (indicated by blue arrowheads) were found only in the VM channels of the control and LIFU groups, possibly due to DSF inhibiting VM function as a metastatic escape route. Moreover, endothelium-dependent microvessels with relatively smooth lumens (Fig. 7A, depicted by brown dashed areas) were distributed throughout the tumor tissues in each group, indicating that the investigation did not affect anti-endothelial cell-dependent angiogenesis. This result was further supported by the mean density data (Additional file 1: Fig. S8), which showed no significant differences among the groups. Given the crucial roles of COL1 and activated MMP-2 in VM formation, we used immunofluorescence and Western blotting to assess the expression levels of these proteins posttreatment. The green fluorescence signals of COL1 and activated MMP-2 in the PFP@PD, PFP@PDM-PEG with or without LIFU, and DSF groups showed varying degrees of attenuation compared to those in the control and LIFU groups. The lowest intensity of both COL1 and activated MMP-2 fluorescence was observed in the PFP@PDM-PEG with LIFU group (Figs. 7B, Additional file 1: Fig. S9-S10). Western blot analysis also confirmed that the tumor tissues in the PFP@PDM-PEG + LIFU group exhibited the lowest of COL1 and activated MMP-2 protein expression levels (Fig. 7C). These findings demonstrate that our treatment approach inhibits tumor growth and metastasis by inhibiting COL1 expression, thereby affecting pro-MMP-2 activation and further inhibiting VM formation in breast cancer-bearing mice.

### In Vivo Biological Safety Evaluation

The in vivo biological safety assessment revealed that the blood biochemistry and routine blood indices remained within normal limits (Additional file 1: Fig. S11). H&E staining of vital organs (Additional file 1: Fig. S12) indicated that none of the treatments had any adverse effects on the organ health of the mice. Preliminary studies confirmed the biosafety of each treatment in vivo, demonstrating that all treatments were well tolerated at the tested levels. Notably, no significant weight loss was observed in the mice following these treatments (Additional file 1: Fig. S13). This finding suggested that neither free DSF nor DSF-loaded nanodroplets induced systemic toxicity, even in the presence of LIFU radiation.

## Conclusion

In this study, we repurposed the FDA-approved antialcoholism drug DSF with a versatile nanoplatform. This platform is enhanced by MMP-2-responsive drug delivery mechanisms and is specifically engineered to increase drug accumulation in tumor regions with dense VM. This was achieved by surface modification with the PEGylated peptide EGPLGVRGK, which is responsive to the trigger MMP-2, leading to effective PEG deshielding. This tailored response to MMP-2 not only facilitates cellular drug uptake but also optimizes drug distribution within the tumor. Furthermore, the integration of image-guided low-intensity focused ultrasound (LIFU) precisely controlled the release of DSF, ensuring its deeper penetration and more uniform dispersion within the tumor. This innovative approach enables controlled drug release while simultaneously inhibiting the expression of COL1. The inhibition of COL1, in turn, hampers the activation of pro-MMP-2, thereby disrupting VM, a critical factor in tumor growth and metastasis. Importantly, these VM-resistant effects of the DSF-loaded nanodroplets contributed to the inhibition of tumor growth and lung metastasis, highlighting their therapeutic potential. The intelligent design of this nanodroplet system, coupled with its biofriendly functionalization, offers a novel and strategic approach to tackling the complex microvascular environment of TNBC. This study not only demonstrates the feasibility of using LIFU irradiation to disrupt such environments but also opens new avenues for effective cancer treatment strategies.

### Supplementary Information

**Additional file 1: Fig. S1.** Drug Loading Efficacy of Nanodroplets with Varying Feeding Ratios of DSF and PLGA-MMP-2-PEG. **Fig. S2.** Detection of conjugation efficiency of the FITC-labelled MMP-2-PEG and PLGA-COOH. **Fig. S3.** HPLC chromatograms of DSF. **Table S1.** Average particle size, zeta potential, drug loading and encapsulation efficiency of PFP@PD and PFP@PDM-PEG. (n=3, mean ± SD). **Fig. S4.** *In vitro *drug release profiles of PFP@PD and PFP@PDM-PEG. (n=3, mean ± SD). **Fig. S5.** Antitumor efficacy of PFP@PDM-PEG in vitro. **Fig. S6.** Representative images of the 4T1 tumors after different treatment at day 14. **Fig. S7.** Representative H&E staining of the liver in each group. The scale bar: 10 μm. **Fig. S8.** Calculation of the mean density of endothelium-dependent microvessels in each group. **Fig. S9.** Calculation of the fluorescence intensity of COL1 in each group. (n=3, t-test, *p < 0.05, **p < 0.01, ***p < 0.001). **Fig. S10.** Calculation of the fluorescence intensity of activated MMP-2 in each group. (n=3, t-test, **p < 0.01, ***p < 0.001, ****p < 0.0001). **Fig. S11.** All mouse hematologic and serum biomedical indices. **Fig. S12.** H&E staining of the vital organs (heart, liver, spleen, lungs, and kidneys). **Fig. S13.** Variations in body weight of 4T1 tumor-bearing mice in various groups.

## Data Availability

The data presented in this study are available from the corresponding author upon reasonable request.

## References

[1] Bissell MJ (1999). Tumor plasticity allows vasculogenic mimicry, a novel form of angiogenic switch. A rose by any other name. Am J Pathol.

[2] Barinaga M (1999). New type of blood vessel found in tumors. Science.

[3] Folberg R (1992). The morphologic characteristics of tumor blood vessels as a marker of tumor progression in primary human uveal melanoma: a matched case-control study. Hum Pathol.

[4] Wei X (2021). Mechanisms of vasculogenic mimicry in hypoxic tumor microenvironments. Mol Cancer.

[5] Maniotis AJ (1999). Vascular channel formation by human melanoma cells in vivo and *in vitro*: vasculogenic mimicry. Am J Pathol.

[6] Stlhammar G, See T, Phillips SS, Grossniklaus HE (2019). Density of PAS positive patterns in uveal melanoma: Correlation with vasculogenic mimicry, gene expression class, BAP-1 expression, macrophage infiltration, and risk for metastasis. Mol Vis.

[7] Ricci-Vitiani L (2010). Tumour vascularization via endothelial differentiation of glioblastoma stem-like cells. Nature.

[8] Mitra D (2020). Phosphorylation of EphA2 receptor and vasculogenic mimicry is an indicator of poor prognosis in invasive carcinoma of the breast. Breast Cancer Res Treat.

[9] Shuai Q, Cao L, Qin Z, Zhang Y, Gu Z, Yang J (2020). VE-cadherin fusion protein substrate enhanced the vasculogenic mimicry capability of hepatocellular carcinoma cells. J Mater Chem B.

[10] Lin PP (2020). Aneuploid circulating tumor-derived endothelial cell (CTEC): a novel versatile player in tumor neovascularization and cancer metastasis. Cells.

[11] Ribatti D, Nico B, Ruggieri S, Tamma R, Simone G, Mangia A (2016). Angiogenesis and Antiangiogenesis in Triple-Negative Breast cancer. Transl Oncol.

[12] Wechman SL, Emdad L, Sarkar D, Das SK, Fisher PB (2020). Vascular mimicry: Triggers, molecular interactions and in vivo models. Adv Cancer Res.

[13] Fathi Maroufi N (2020). Vascular mimicry: changing the therapeutic paradigms in cancer. Mol Biol Rep.

[14] Zheng N, Zhang S, Wu W, Zhang N, Wang J (2021). Regulatory mechanisms and therapeutic targeting of vasculogenic mimicry in hepatocellular carcinoma. Pharmacol Res.

[15] Maroufi NF (2020). Inhibitory effect of melatonin on hypoxia-induced vasculogenic mimicry via suppressing epithelial-mesenchymal transition (EMT) in breast cancer stem cells. Eur J Pharmacol.

[16] Maroufi NF (2022). Effect of Apatinib plus melatonin on vasculogenic mimicry formation by cancer stem cells from breast cancer cell line. Breast Cancer.

[17] Luo Q (2020). Vasculogenic mimicry in carcinogenesis and clinical applications. J Hematol Oncol.

[18] Lu C, Li X, Ren Y, Zhang X (2021). Disulfiram: a novel repurposed drug for cancer therapy. Cancer Chemother Pharmacol.

[19] Skrott Z (2017). Alcohol-abuse drug disulfiram targets cancer via p97 segregase adaptor NPL4. Nature.

[20] Wang Z (2017). Poly lactic-co-glycolic acid controlled delivery of disulfiram to target liver cancer stem-like cells. Nanomedicine.

[21] Zhang X (2019). Induction of autophagy-dependent apoptosis in cancer cells through activation of ER stress: an uncovered anti-cancer mechanism by anti-alcoholism drug disulfiram. Am J Cancer Res.

[22] Nasrollahzadeh A (2021). Anti-proliferative activity of disulfiram through regulation of the AKT-FOXO axis: a proteomic study of molecular targets. Biochim Biophys Acta Mol Cell Res.

[23] Wang DR, Sato M, Li LN, Miura M, Kojima N, Senoo H (2003). Stimulation of pro-MMP-2 production and activation by native form of extracellular type I collagen in cultured hepatic stellate cells. Cell Struct Funct.

[24] Kim IY, Jeong SJ, Kim ES, Kim SH, Moon A (2007). Type I collagen-induced pro-MMP-2 activation is differentially regulated by H-Ras and N-Ras in human breast epithelial cells. J Biochem Mol Biol.

[25] Itoh Y, Seiki M (2004). MT1-MMP: an enzyme with multidimensional regulation. Trends Biochem Sci.

[26] Haas TL, Davis SJ, Madri JA (1998). Three-dimensional type I collagen lattices induce coordinate expression of matrix metalloproteinases MT1-MMP and MMP-2 in microvascular endothelial cells. J Biol Chem.

[27] Ellerbroek SM, Wu YI, Overall CM, Stack MS (2001). Functional interplay between type I collagen and cell surface matrix metalloproteinase activity. J Biol Chem.

[28] Liang X (2017). Rictor regulates the vasculogenic mimicry of melanoma via the AKT-MMP-2/9 pathway. J Cell Mol Med.

[29] Chen D, Dou QP (2008). New uses for old copper-binding drugs: converting the pro-angiogenic copper to a specific cancer cell death inducer. Expert Opin Ther Targets.

[30] Fasehee H (2016). Delivery of disulfiram into breast cancer cells using folate-receptor-targeted PLGA-PEG nanoparticles: in vitro and in vivo investigations. J Nanobiotechnology.

[31] Bourboulia D, Stetler-Stevenson WG (2010). Matrix metalloproteinases (MMPs) and tissue inhibitors of metalloproteinases (TIMPs): Positive and negative regulators in tumor cell adhesion. Semin Cancer Biol.

[32] Zhang X (2023). An MMP-2 sensitive and reduction-responsive prodrug amphiphile for actively targeted therapy of cancer by hierarchical cleavage. Chem Commun (Camb).

[33] Miki K (2021). MMP-2-Activatable Photoacoustic Tumor Imaging Probes Based on Al- and Si-Naphthalocyanines. Bioconjug Chem.

[34] Ni R, Zhu J, Xu Z, Chen Y (2020). A self-assembled pH/enzyme dual-responsive prodrug with PEG deshielding for multidrug-resistant tumor therapy. J Mater Chem B.

[35] Kong L, Campbell F, Kros A (2019). DePEGylation strategies to increase cancer nanomedicine efficacy. Nanoscale Horiz.

[36] Mishra S, Webster P, Davis ME (2004). PEGylation significantly affects cellular uptake and intracellular trafficking of non-viral gene delivery particles. Eur J Cell Biol.

[37] Gong Y (2016). Low-intensity focused ultrasound mediated localized drug delivery for liver tumors in rabbits. Drug Deliv.

[38] Olsman M (2020). Ultrasound-mediated delivery enhances therapeutic efficacy of MMP sensitive liposomes. J Control Release.

[39] Li H (2019). Ultrasound-enhanced delivery of doxorubicin-loaded nanodiamonds from pullulan-all-trans-retinal nanoparticles for effective cancer therapy. ACS Appl Mater Interfaces.

[40] Wang C (2017). Disulfiram-loaded porous PLGA microparticle for inhibiting the proliferation and migration of non-small-cell lung cancer. Int J Nanomedicine.

[41] Fasehee H, Ghavamzadeh A, Alimoghaddam K, Ghaffari SH, Faghihi S (2017). A Comparative cytotoxic evaluation of disulfiram encapsulated PLGA nanoparticles on MCF-7 Cells. Int J Hematol Oncol Stem Cell Res.

[42] Choi JH, Lee JS, Park KM, Bae JW, Lee Y, Park KD (2017). Multi-layered nanogels with MMP-sheddable PEG masks: preparation and promotion of tumor cell uptake by controlling surface characteristics. Colloids Surf B Biointerfaces.

[43] Hatakeyama H (2011). Systemic delivery of siRNA to tumors using a lipid nanoparticle containing a tumor-specific cleavable PEG-lipid. Biomaterials.

[44] Sun W, Li Z, Zhou X, Yang G, Yuan L (2019). Efficient exosome delivery in refractory tissues assisted by ultrasound-targeted microbubble destruction. Drug Deliv.

[45] Yang C (2020). Dual ultrasound-activatable nanodroplets for highly-penetrative and efficient ovarian cancer theranostics. J Mater Chem B.

[46] Zhang L (2019). Mitochondria-targeted and ultrasound-activated nanodroplets for enhanced deep-penetration sonodynamic cancer therapy. ACS Appl Mater Interfaces.

[47] Zhu L (2018). Peptide-functionalized phase-transformation nanoparticles for low intensity focused ultrasound-assisted tumor imaging and therapy. Nano Lett.

