# Supplementary material for: LIFU/MMP-2 dual-responsive release of repurposed drug disulfiram from nanodroplets for inhibiting vasculogenic mimicry and lung metastasis in triple-negative breast cancer
Source: J Nanobiotechnology. 2024 Apr 25;22:209. doi: 10.1186/s12951-024-02492-7 (PMC11046851; doi:10.1186/s12951-024-02492-7)
Supplement: Supplementary file 1 — Additional file 1: Fig. S1. Drug Loading Efficacy of Nanodroplets with Varying Feeding Ratios of DSF and PLGA-MMP-2-PEG. Fig. S2. Detection of conjugation efficiency of the FITC-labelled MMP-2-PEG and PLGA-COOH. Fig. S3. HPLC chromatograms of DSF. Table S1. Average particle size, zeta potential, drug loading and encapsulation efficiency of PFP@PD and PFP@PDM-PEG. (n=3, mean ± SD). Fig. S4. In vitro drug release profiles of PFP@PD and PFP@PDM-PEG. (n=3, mean ± SD). Fig. S5. Antitumor efficacy of PFP@PDM-PEG in vitro. Fig. S6. Representative images of the 4T1 tumors after different treatment at day 14. Fig. S7. Representative H&E staining of the liver in each group. The scale bar: 10 μm. Fig. S8. Calculation of the mean density of endothelium-dependent microvessels in each group. Fig. S9. Calculation of the fluorescence intensity of COL1 in each group. (n=3, t-test, *p < 0.05, **p < 0.01, ***p < 0.001). Fig. S10. Calculation of the fluorescence intensity of activated MMP-2 in each group. (n=3, t-test, **p < 0.01, ***p < 0.001, ****p < 0.0001). Fig. S11. All mouse hematologic and serum biomedical indices. Fig. S12. H&E staining of the vital organs (heart, liver, spleen, lungs, and kidneys). Fig. S13. Variations in body weight of 4T1 tumor-bearing mice in various groups. [file 12951_2024_2492_MOESM1_ESM.docx]

**The following is the Supplementary data to this article.**

**
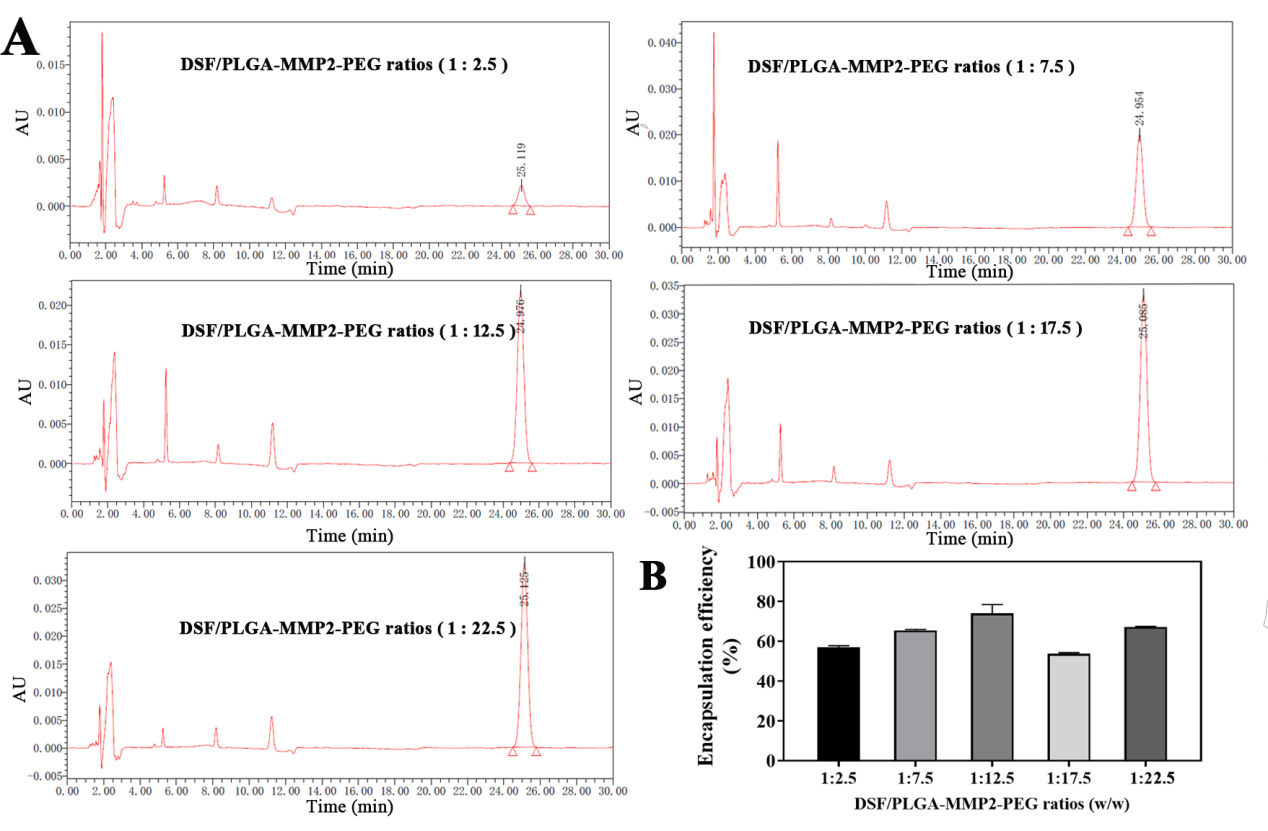
**

**Fig. S1. Drug Loading Efficacy of Nanodroplets with Varying Feeding Ratios of DSF and PLGA-MMP-2-PEG.** (A) HPLC chromatograms of DSF Loading in nanodroplets. (B) Quantitative analysis of drug loading efficacy among nanodroplets with different feeding ratios.

**
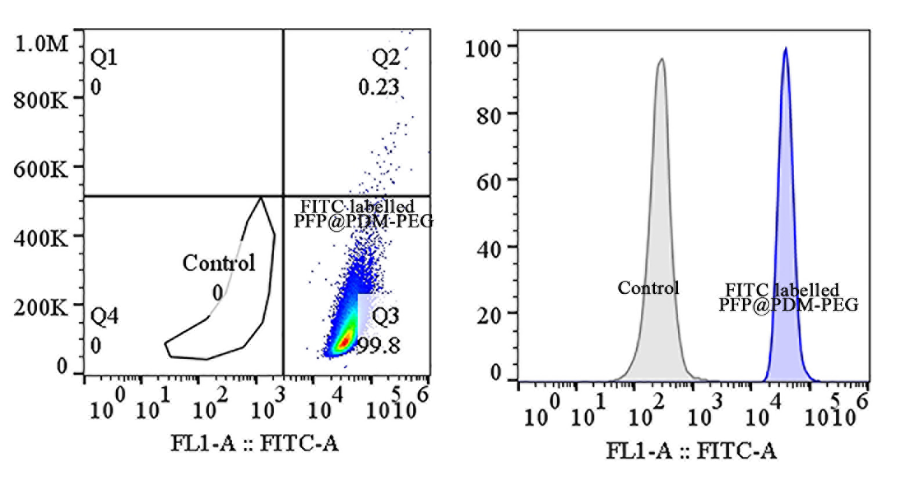
**

**Fig. S2 Detection of conjugation efficiency of the FITC-labelled MMP-2-PEG and PLGA-COOH.**

**

**

**Fig. S3 HPLC chromatograms** **of DSF.** (A) HPLC chromatograms of free DSF with the standard curve. (B) HPLC chromatograms of DSF loading in PFP@PDM-PEG. (C) HPLC chromatograms of DSF loading in PFP@PD.

| nanodroplets | Average particle size (nm) | Zeta potential (mV) | Drug loading (%) | Encapsulation efficiency (%) |
| --- | --- | --- | --- | --- |
| PFP@PDM-PEG | 343.5 ± 119.2 | -9.3 ± 3.5 | 6.8 ± 0.1 | 75.7 ± 15.2 |
| PFP@PD | 305.5 ± 96.3 | -5.6 ± 3.7 | 7.1 ± 0.0 | 77.6 ± 6.3 |

**Table S1 Average particle size, zeta potential, drug loading and encapsulation efficiency of PFP@PD and PFP@PDM-PEG.** (n=3, mean ± SD).





**Fig. S4 *In vitro* drug release profiles of PFP@PD and PFP@PDM-PEG.** (n=3, mean ± SD).





**Fig. S5 Antitumor efficacy of PFP@PDM-PEG in vitro.** **(A)** Cell viability and **(B-D)** Curves of IC50 of free DSF, PFP@PDM-PEG without MMP-2, and PFP@PDM-PEG with MMP-2 on 4T1 cells. (n=3, t-test, ***p* < 0.01, *****p* <0.0001). (C) Flow analysis of apoptosis in various groups. (D) Quantitative analysis of apoptosis. (n=3, t-test, ****p* < 0.001, *****p* < 0.0001).

**
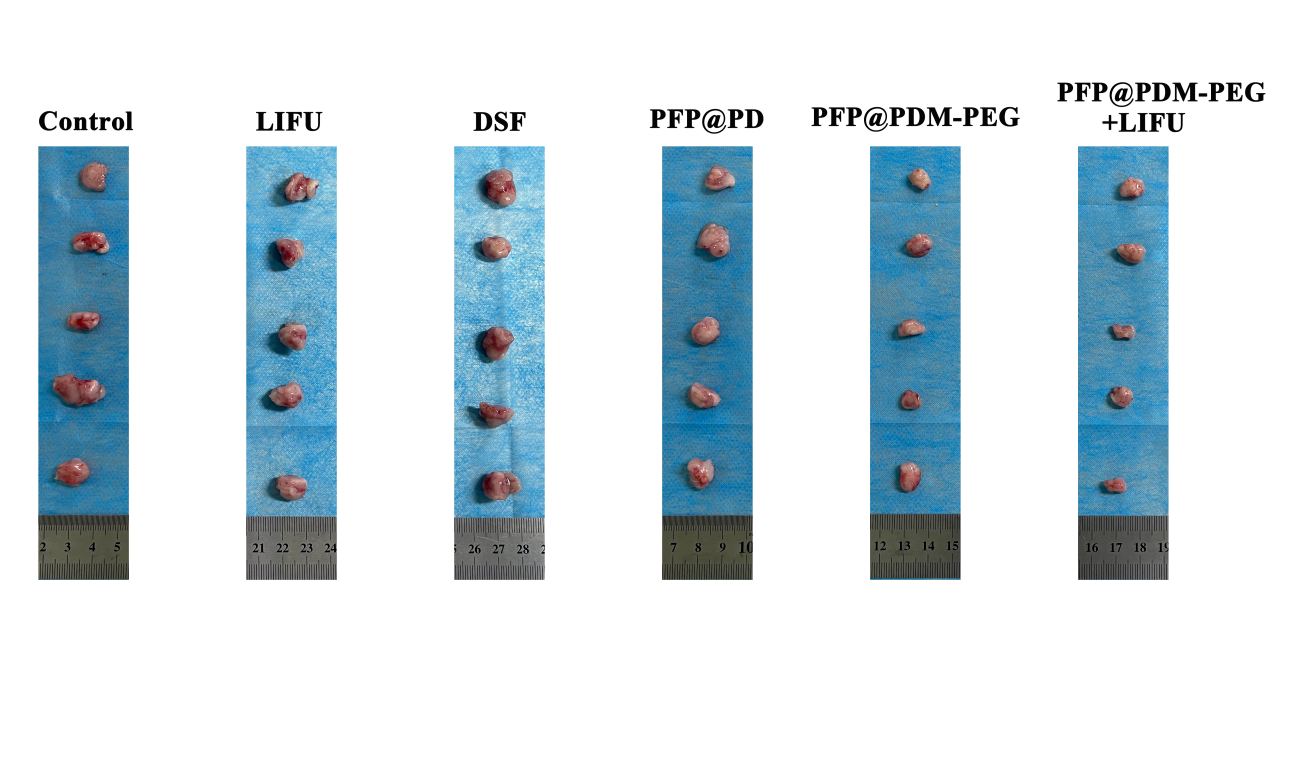
**

**Fig. S6 Representative images of the 4T1 tumors after different treatment at day 14.**

**
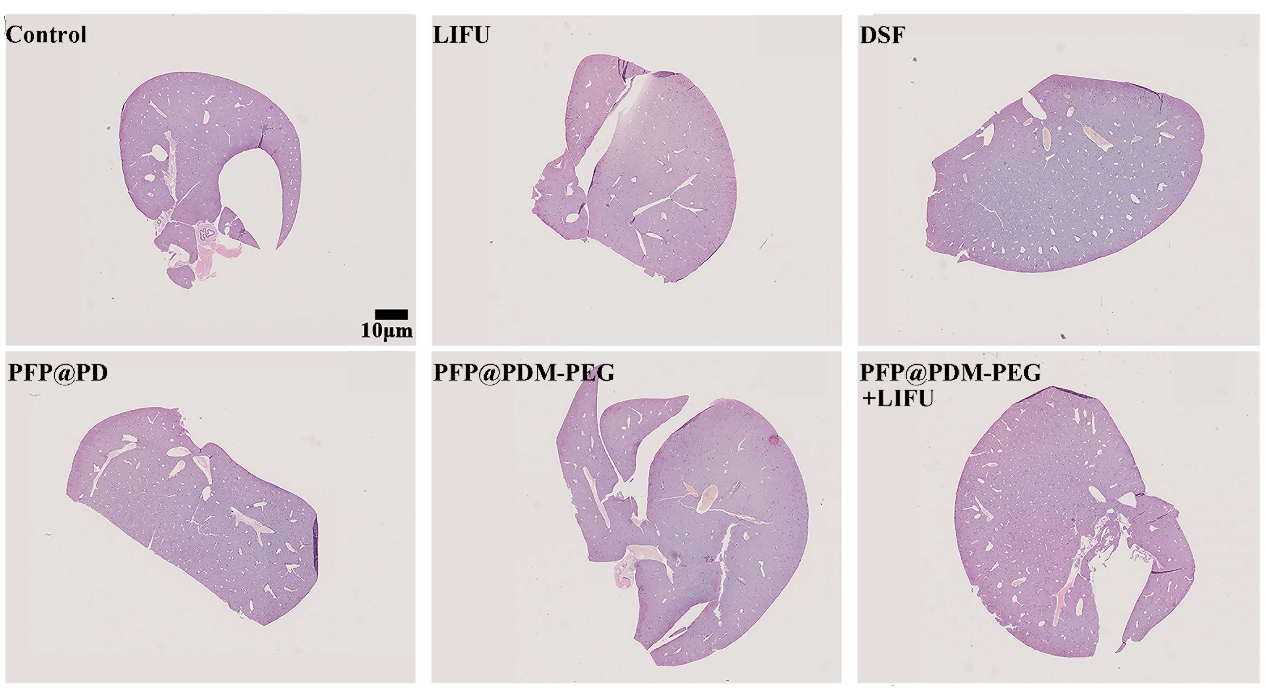
**

**Fig. S7 Representative H&E staining of the liver in each group.** The scale bar: 10 μm.

**
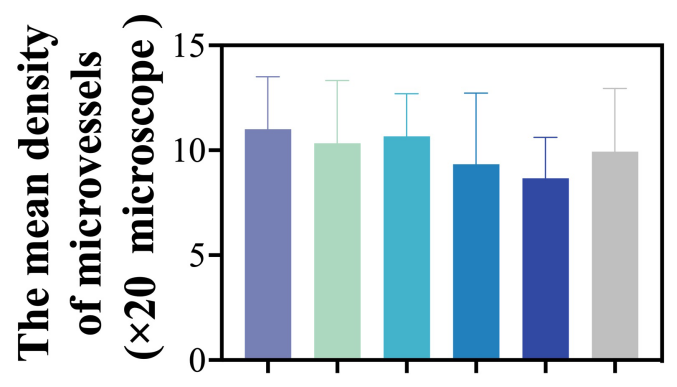
**

**Fig. S8 Calculation of the mean density of endothelium-dependent microvessels in each group.**


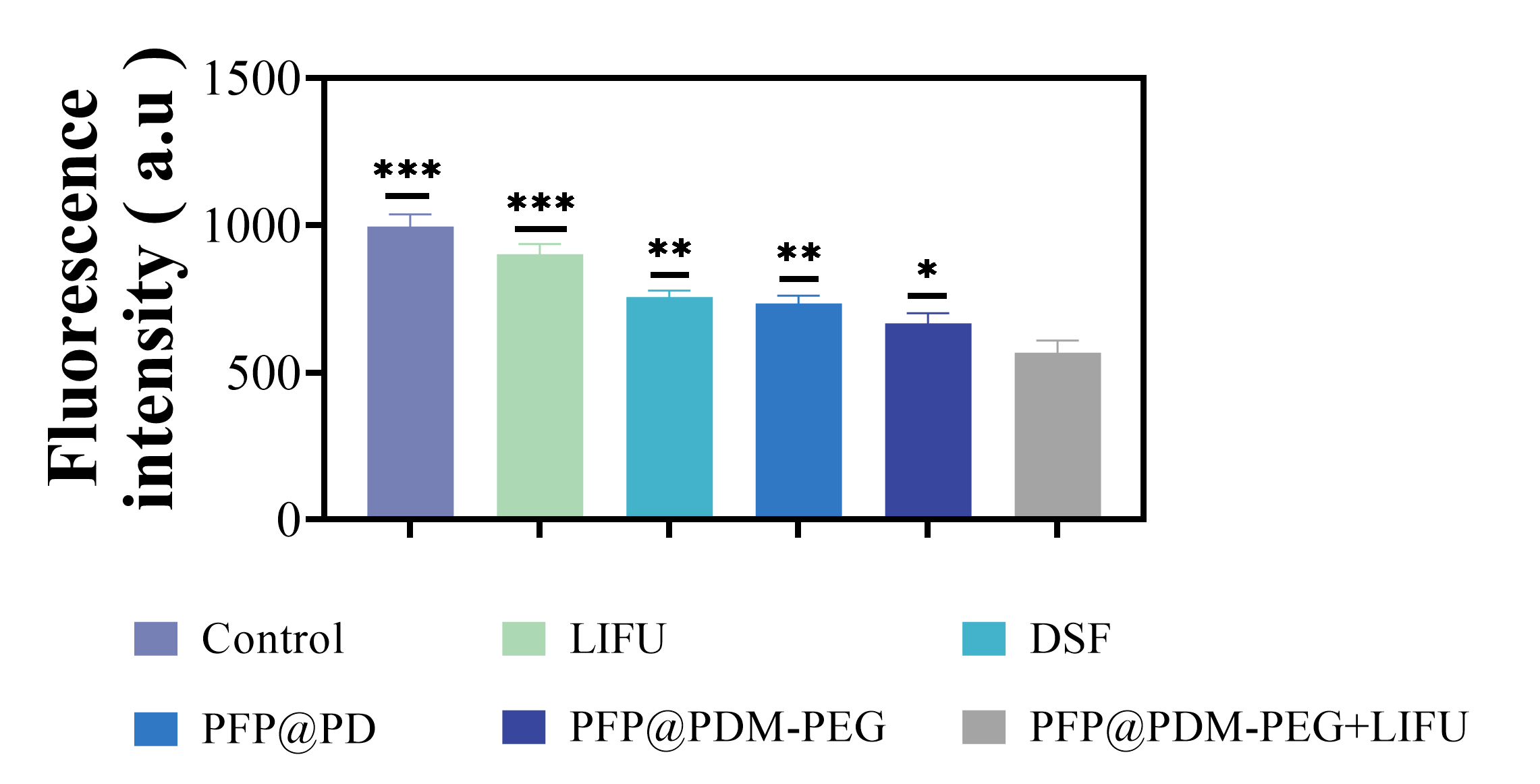


**Fig. S9 Calculation of the fluorescence intensity of COL1 in each group.** (n=3, t-test, **p* < 0.05, ***p* < 0.01, ****p* < 0.001).


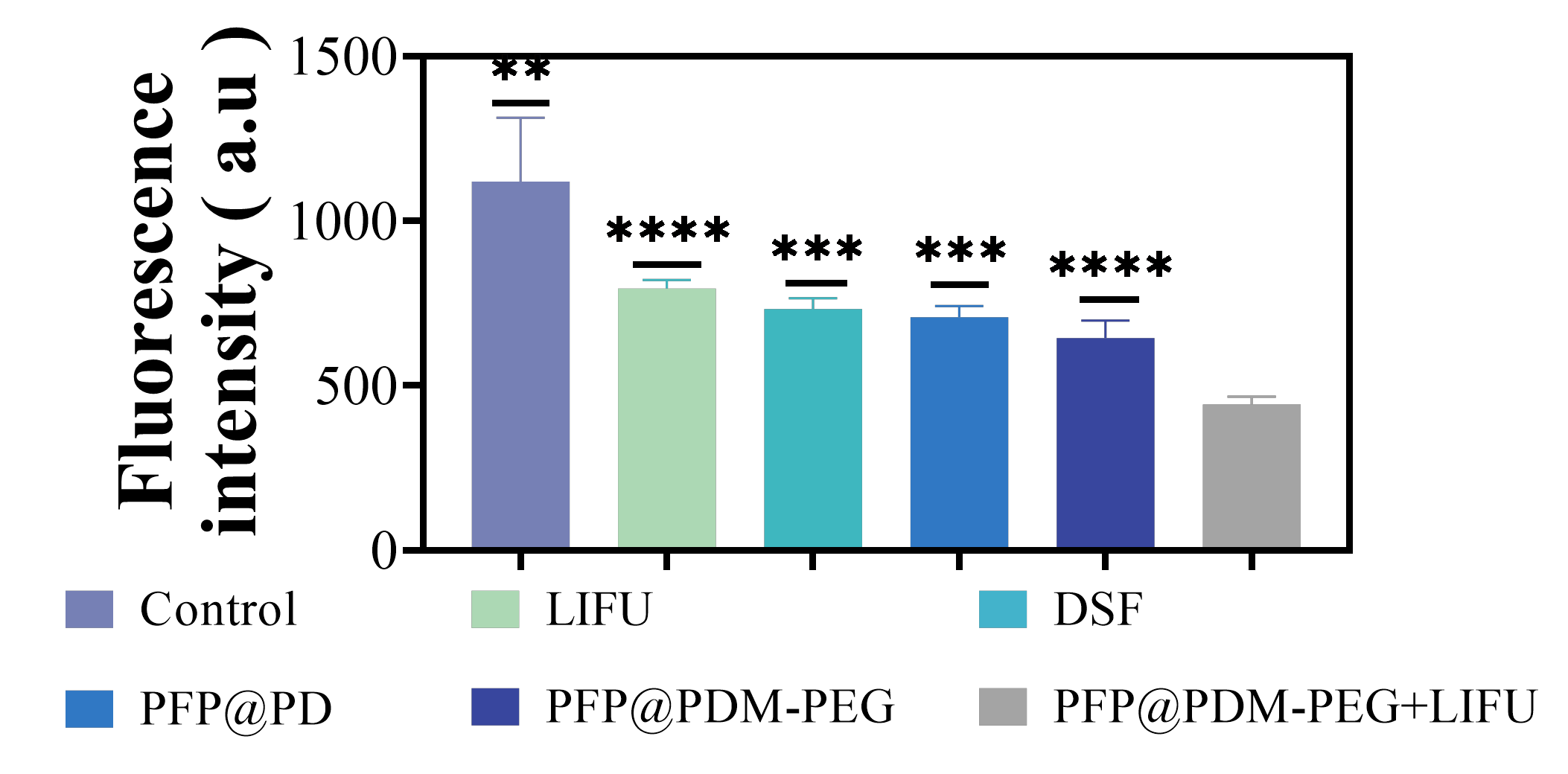


**Fig. S10 Calculation of the fluorescence intensity of activated MMP-2 in each group.** (n=3, t-test, ***p* < 0.01, ****p* < 0.001, *****p* < 0.0001).


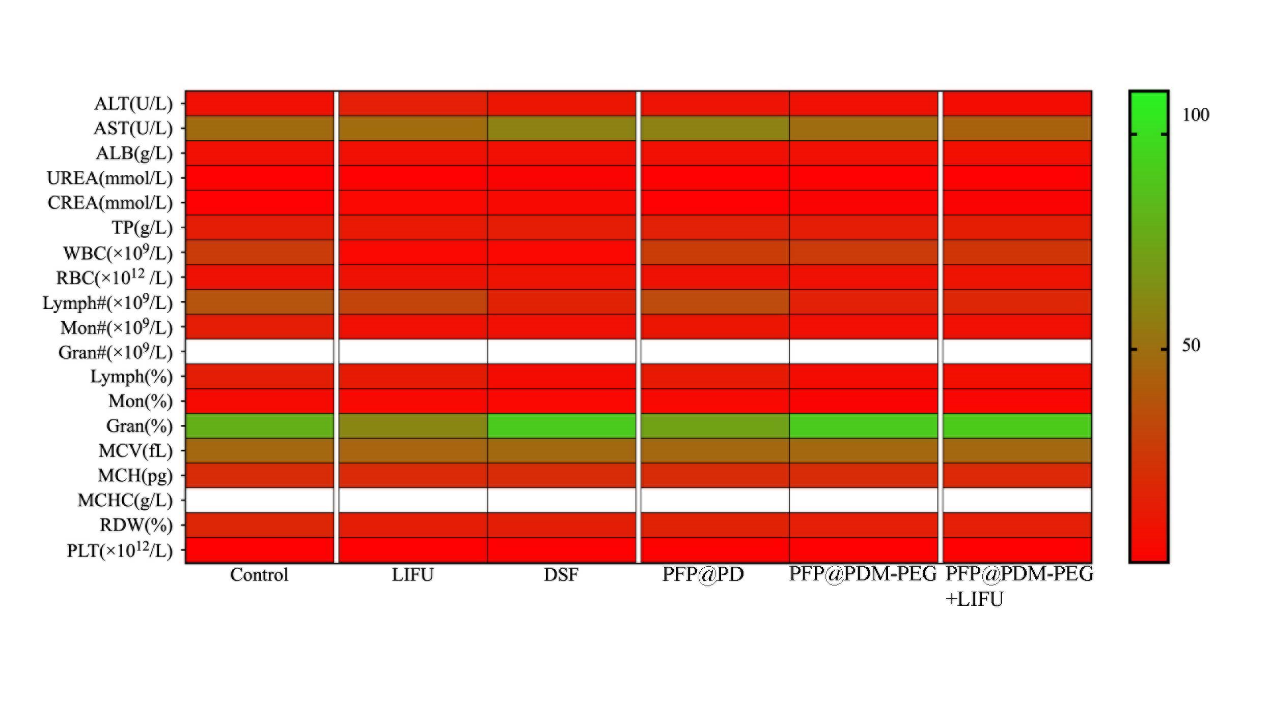


**Fig. S11** **All mouse hematologic and serum biomedical indices.**


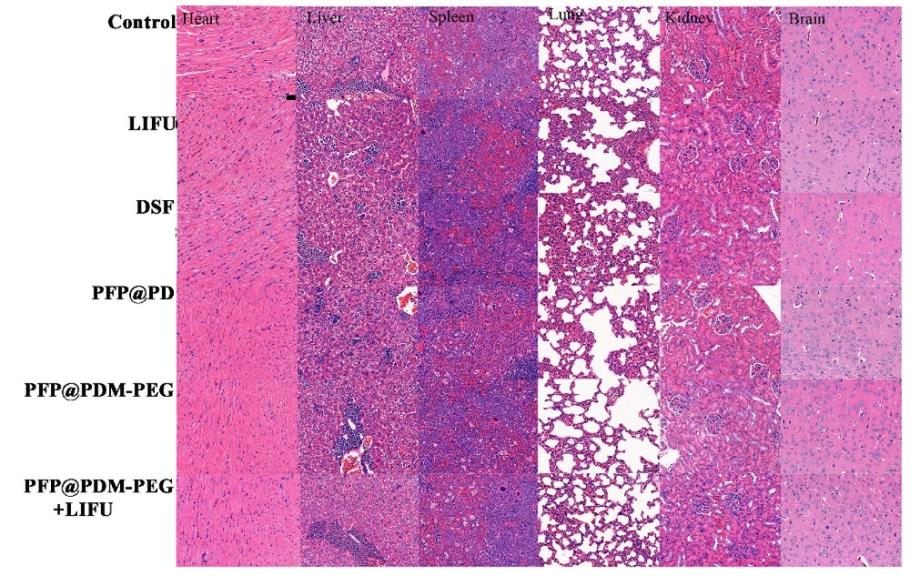


**Fig. S12 H&E staining of the vital organs (heart, liver, spleen, lungs, and kidneys).**


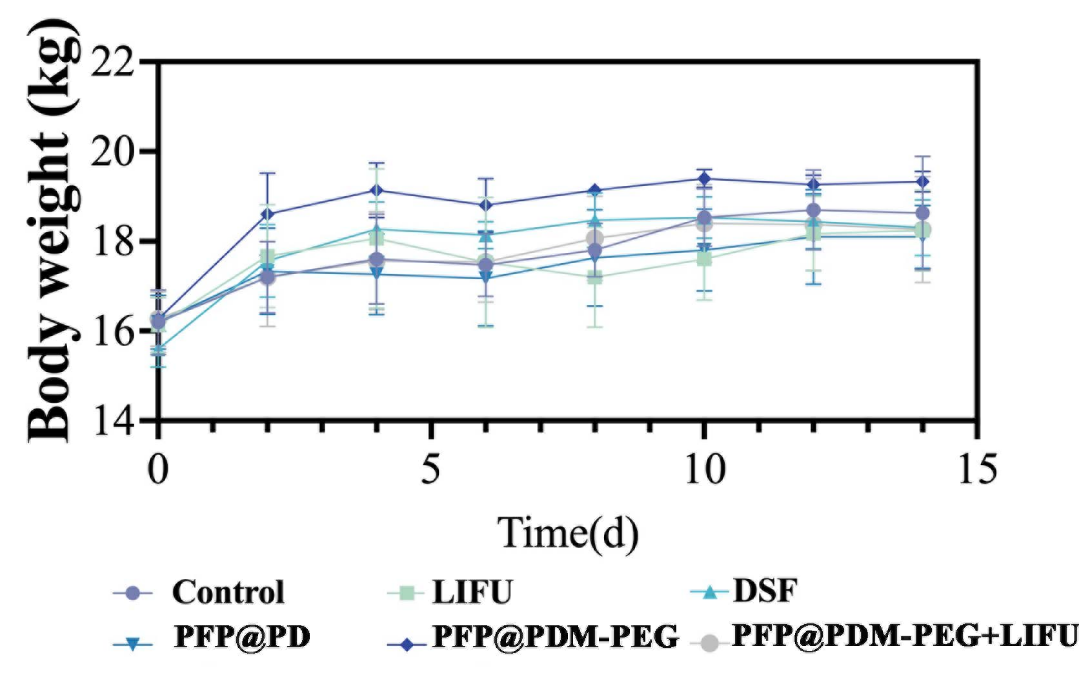


**Fig. S13 Variations in body weight of 4T1 tumor-bearing mice in various groups.**
